# Cysteine (C)-X-C Receptor 4 Undergoes Transportin 1-Dependent Nuclear Localization and Remains Functional at the Nucleus of Metastatic Prostate Cancer Cells

**DOI:** 10.1371/journal.pone.0057194

**Published:** 2013-02-28

**Authors:** Ayesha S. Don-Salu-Hewage, Siu Yuen Chan, Kathleen M. McAndrews, Mahandranauth A. Chetram, Michelle R. Dawson, Danaya A. Bethea, Cimona V. Hinton

**Affiliations:** 1 Center for Cancer Research and Therapeutic Development, Clark Atlanta University, Atlanta, Georgia, United States of America; 2 Department of Biological Sciences, Clark Atlanta University, Atlanta, Georgia, United States of America; 3 Department of Paediatrics and Adolescent Medicine, Queen Mary Hospital, University of Hong Kong, Hong Kong, PRC; 4 School of Chemical & Biomolecular Engineering, Georgia Institute of Technology, Atlanta, Georgia, United States of America; University of Kentucky College of Medicine, United States of America

## Abstract

The G-protein coupled receptor (GPCR), Cysteine (C)-X-C Receptor 4 (CXCR4), plays an important role in prostate cancer metastasis. CXCR4 is generally regarded as a plasma membrane receptor where it transmits signals that support transformation, progression and eventual metastasis. Due to the central role of CXCR4 in tumorigenesis, therapeutics approaches such as antagonist and monoclonal antibodies have focused on receptors that exist on the plasma membrane. An emerging concept for G-protein coupled receptors is that they may localize to and associate with the nucleus where they retain function and mediate nuclear signaling. Herein, we demonstrate that CXCR4 associated with the nucleus of malignant prostate cancer tissues. Likewise, expression of CXCR4 was detected in nuclear fractions among several prostate cancer cell lines, compared to normal prostate epithelial cells. Our studies identified a nuclear pool of CXCR4 and we defined a nuclear transport pathway for CXCR4. We reveal a putative nuclear localization sequence (NLS), ‘RPRK’, within CXCR4 that contributed to nuclear localization. Additionally, nuclear CXCR4 interacted with Transportinβ1 and Transportinβ1-binding to CXCR4 promoted its nuclear translocation. Importantly, G_αi_ immunoprecipitation and calcium mobilization studies indicated that nuclear CXCR4 was functional and participated in G-protein signaling, revealing that the nuclear pool of CXCR4 retained function. Given the suggestion that functional, nuclear CXCR4 may be a mechanism underlying prostate cancer recurrence, increased metastatic ability and poorer prognosis after tumors have been treated with therapy that targets plasma membrane CXCR4, these studies addresses a novel mechanism of nuclear signaling for CXCR4, a novel mechanism of clinical targeting, and demonstrate an active nuclear pool that provides important new information to illuminate what has been primarily clinical reports of nuclear CXCR4.

## Introduction

Prostate cancer (PCa) is the second leading cause of increased cancer incidence and cancer-related deaths among men in the United States [1], [2]. Despite treatment, the high mortality rates in PCa are attributed to metastasis, which is the main obstacle in PCa treatment [3]. Several molecules and mechanisms contribute to cancer cell metastasis. For instance, chemoattractant cytokines (chemokines) enhances the metastatic potential of PCa by binding and activating a family of G-protein coupled receptors (GPCRs) [4], [5], [6], [7] that initiate signals to enhance cell adhesion, invasion and movement, and subsequently, tumor survival at the new site of metastasis. GPCRs constitute the largest family of transmembrane plasma membrane (PM) receptors [8]. In conventional GPCR signaling, receptors are localized to the PM and influence the activity of PM-localized enzymes, ion channels, and/or second messengers. Their activation by an appropriate ligand triggers signaling through G-protein alpha (G_α_) and/or beta-gamma (G_βγ_) subunits [9], leading to context-dependent outcomes, which may positively and/or negatively regulate the activity of effector molecules in signaling cascades within the cell [10], [11]. Additionally, activated GPCRs also trigger a series of molecular interactions that allow for feedback regulation of G-protein coupling and receptor endocytosis to attenuate receptor signals [12], [13], [14], [15], [16], [17], [18]. Müller *et al*. initially described the involvement of chemokine GPCR receptors in cancer metastasis [19] and Akashi *et al.* reported that the chemokine GPCR, CXCR4, was highly expressed in human malignant PCa compared to normal prostate [20]. Numerous studies have documented the involvement of CXCR4 in key steps of PCa metastasis: (i) signaling; [21], [22]; (ii) invasion and migration [23]; and (iii) the establishment of a vascular network [24]. Hence, several therapeutics for cancer cell metastasis have been designed to antagonize CXCR4-mediated signaling [25], [26]. In conventional CXCR4 signaling, stromal cell-derived factor 1 alpha (SDF1α) is the exclusive ligand for CXCR4 [27], which leads to activation of pathways makes this receptor favorable to tumorigenesis: (i) G-protein coupled receptor (GPCR) signaling; (ii) PI3K/AKT; (iii)MAPK; (iv) JAK/STAT; (v) Src kinase and (vi) HER2 [28], [29], [30].

Interestingly, GPCRs have been detected in subcellular organelles distinct from its classical PM location [31]. These organelles include the Golgi apparatus [32], endoplasmic reticulum [33], the cytoskeleton [34] and the nucleus/nuclear membrane [35]. Hanyaloglu and von Zastrow postulated that default recycling of GPCRs by endosomes may contribute to enhanced re-delivery of GPCRs to the PM, or to alternate organelles within the cell, without destroying their signaling capacity [36]. Nevertheless, these alternately-localized GPCR receptors reveal a new level of complexity that may be important in modulating their function. An increasing number of GPCRs have been observed within the nucleus or nuclear membrane, such as lysophosphatidic acid receptors, metabotropic glutamate receptors, platelet-activating factor receptors, angiotensin 2 type I receptors, prostaglandin receptors, endothelin receptors, gonadotropin releasing hormone type I receptor [37] and *β*-adrenergic receptors [38], [39], [40], [41], [42], [43], [44]. Nuclear GPCRs have been suggested to regulate a number of physiological processes, including cell proliferation, survival, inflammatory responses, tumorgenesis, DNA synthesis and transcription [43], [45], [46], [47], [48], [49], [50]. Nuclear GPCRs may be constitutively active, or activated by internal, newly synthesized ligands that are bound for secretion [51]. Subsequently, classical second messenger signaling pathways, such as adenylyl cyclase-induced Protein Kinase A (PKA) activation [38], phospholipase-induced release of intranuclear calcium, diacyglycerol-induced Protein Kinase C (PKC) [39], [52], ERK1/2, p38 MAP Kinases and Protein Kinase B (PKB) [49], [50] have been shown to be activated by nuclear GPCRs.

Nuclear localization of proteins is dictated by nuclear import and export through nuclear pore complexes [53]. Small proteins (<30–50 kDa) can pass through the nuclear pore by free diffusion; however, most cargo proteins require active transport to enter the nucleus [54]. Larger proteins use active transport mechanisms, which require assistance by transport proteins [55], [56], [57]. Many proteins targeted to the nucleus contain a classical nuclear localization signal (NLS) that is recognized by a heterodimeric import receptor comprised of importin alpha and importin beta. Many of these receptors directly recognize cargo proteins and target them directly to the nuclear pore [58]. In the case of this large family, the targeting signals within the cargo proteins are often not well-defined [59]. Each protein that localizes to the nucleus must possess a functional NLS or is required to bind to cargo proteins which possess a NLS(s). Importin alpha recognizes the NLS in the cargo protein while importin beta targets the import complex to the nuclear pore [58], [60]. Importin beta is part of a larger family of transport receptors often termed importins/exportins [59].

While a putative NLS has been identified in CXCR4 [61], the function of this nuclear targeting signal in the context of CXCR4 has not been examined. A distinct importin-dependent transport pathway has been implicated in the transport of C-C chemokine receptor type 2 (CCR2) [62]. Favre *et al*. found that an engineered, HA-tagged CCR2 associated with a member of the importin family of nuclear transport receptors, Transportinβ1 (TRN1), in a CCR2-null cell line [62]. An interaction of CCR2 with TRN1 was required to detect CCR2 in nuclear fractions, suggesting that CCR2 transported to the nucleus via TRN1 [62]. TRN1 has been implicated in GPCR internalization and desensitization [63]. Furthermore, TRN1 serves as a receptor for NLS-null proteins in NLS-containing cargo substrates [64], making it an essential protein for import through the nuclear pore complex [65]. Taken together, these studies suggest that both the classical nuclear import machinery and TRN1 are candidates that play a role in CXCR4 nuclear translocation [66].

Nuclear CXCR4 protein expression has been observed in malignant hepatocellular, colorectal, renal cell and nasopharyngeal carcinomas [67], [68], [69], [70]. These studies, however, were reported as clinical observations, and failed to investigate the mechanisms of CXCR4 localization or any biological function associated with the nuclear receptor. These data are consistent with reports that have demonstrated functional GPCRs associated with the nucleus, and further contribute to ongoing cancer therapeutic interventions against CXCR4. Importantly, a functional nuclear CXCR4 may contribute to PCa relapse despite current antagonists and monoclonal antibodies against PM-bound CXCR4 and may not be designed to cross the PM, which would be required to antagonize active CXCR4 at the nucleus. Furthermore, identification of transport pathways required for nuclear localization of CXCR4 may reveal additional targets for therapeutic development to hinder prostate cancer metastasis and improve patient survival.

## Materials and Methods

### Cell Culture, Antibodies and Reagent Conditions

PC3, DU145, 22RV1 human prostate cancer cell lines (PCa), RWPE1 human prostate cell line and 293T human embryonic kidney cell line were obtained from American Type Culture Collection (ATCC). PC3, DU145, 22RV1 and 293T cells were maintained in complete RPMI media: RPMI 1640 containing 10% fetal bovine serum (FBS), 1% non-essential amino acids and 1% antibiotic-antimycotic at 37°C in 5% CO_2_. RWPE1 cells were maintained in keratinocyte serum-free medium (KSFM) containing 50 mg/ml gentamycin, 0.05 mg/ml bovine pituitary extract (BPE), and 5 ng/ml epidermal growth factor (Invitrogen) at 37°C in 5% CO_2_. All cells were maintained at 60% to 80% confluency. PC3 cells were originally isolated from a prostate vertebral metastasis, while DU145 cells were obtained from prostate brain metastasis. 22RV1 cells were from a human prostate carcinoma epithelial cell line derived from a xenograft that was serially propagated in mice, and RWPE1 cells were isolated from normal human prostate epithelium. Cell culture supplies and kanamycin sulfate (61-176-RG) were from MediaTech; SDF1α (300-28A) was from PeproTech. The following reagents and human antibodies were from Cell Signaling: 10× cell lysis buffer (9803), mouse anti-rabbit IgG (5127), anti-CD44 (156-3C11), anti-GFP (2956S) and anti-G_αi_ (5290). Anti-CXCR4 (MAB172) was from R&D Systems. Anti-Topoisomerase1 (SC-271285), anti-Lamin A/C (SC-20681), Fusin (H-118)-CXCR4 (SC-9046), Fusin (4G10)-CXCR4 (SC- 53534), anti-Fibronectin IgG2B (SC-271098), anti-GFP (sc-9996), Protein A/G Plus-Agarose beads (SC-2003), anti-Karyopherinβ2 (SC-166127), Karyopherinβ2 siRNA (h) (SC-35737) and DAPI (SC-3598) were from Santa Cruz Biotech. NE-PER Nuclear and Cytoplasmic Extraction Kit (78833), Protease Inhibitor Cocktail Kit (78410) and Halt TM Phosphate Inhibitor Cocktail (78420) were from Thermo Scientific. Anti-αTubulin (T5168), Anti-βActin (A5441) Triton X-100 (T8532) and propidium iodide (P4170) were from Sigma-Aldrich. Prostate disease spectrum tissue array (PR8011) was purchased from Biomax. JetPRIME® Polypus transfection reagent (114-07) was from VWR International. Nonidet P-40 Substitute (M158) was from BioExpress and FluoForte Calcium Assay Kit (ENZ-51016) was from Enzo Life Sciences.

### Characterization of CXCR4 IgG2B (R&D systems) Antibody

Specificity of anti-human CXCR4 mouse monoclonal antibody (R&D Systems) to CXCR4 protein was determined by immunoprecipitation and western blot analysis using CXCR4-positive PC3 and CXCR4-null 293T whole cell lysates. Briefly, PC3 and 293T cells (5×10^6^) were grown on 100 mm dishes in complete media overnight, followed by incubation in RPMI only (serum-starvation) for 24 hrs. Cell were washed with 1× phosphate-buffered saline (PBS) and harvested in 1× Cell Signaling lysis buffer. Equal protein concentrations were estimated by Bradford assay (BioRad) and equal amounts were assessed for western blot analysis with CXCR4-IgG2B mouse monoclonal antibody or 1 mg of supernatant was immunoprecipitated (IP) with CXCR4-IgG2B mouse monoclonal antibody or Fibronectin-IgG2B mouse monoclonal antibody overnight at 4°C (Santa Cruz; 1 µg per 250 µg of protein), followed by incubation with Protein A/G Plus-Agarose beads for 2 hrs at 4°C. Protein-bound agarose beads were separated from lysates by a series of 3 washes with 1× PBS and centrifugation (max speed/2 min/room temperature [RT]). Beads in Lammelli buffer were separated by 10% SDS-PAGE, transferred to polyvinylidenefluoride (PVDF) membranes and probed for CXCR4-IgG2B (1∶1000). To confirm that PC3 cells expressed Fibronectin, 25 µg of whole cell lysate was harvested for western blot analysis. Beta-actin was used as a loading control.

### Immunohistochemistry (IHC)

IHC analysis was performed on a prostate disease spectrum tissue array (Biomax) ranging from normal to high grade metastatic tissues. The array consisted of 80 total tissue cores including adenocarcinoma, metastatic, hyperplasia, chronic inflammation, adjacent normal tissue and normal tissue. Each individual core had a diameter of 1.5 mm and a thickness of 0.5 µm. Briefly, formalin-fixed, paraffin-embedded specimens were retrieved in washes of xylene, ethanol, and antigen retrieval solution, pH 6.0, (Biocare Medical) at 125°C for 30 sec. Specimens were neutralized in 0.3% hydrogen peroxide for 15 min at room temperature (RT), washed with 1× PBS in a humidified chamber and blocked with blocking solution (5% normal goat serum/Tris-buffered saline/Tween-20; TBST) for 30 min. CXCR4 was detected with a mouse anti-human CXCR4 monoclonal antibody (R&D Systems; 1∶1000) in blocking solution overnight at 4°C, followed by a biotinylated affinity purified goat anti-mouse IgG (H+L) secondary antibody (Vector Laboratories;1∶1000), in blocking solution for 30 min at RT. Specimens were washed thoroughly between incubations, developed in diaminobenzidine (Vector Laboratories) for 3 min at RT, and counterstained with Meyer's hematoxylin using standard techniques. A negative control tissue sample was prepared by incubating in biotinylated affinity purified goat anti-mouse IgG (H+L) antibody, only, as described above. The specimens were analyzed and photographed by Dr. Dezhi Wang [71] at the Center for Metabolic Bone Disease Core Laboratory, UAB School of Medicine, Birmingham, Alabama. The distribution of positive cells for CXCR4 was recorded to portray the diffuse or focal nature of the positive cells as sporadic (positive cells <5%); focal (positive cells >11% but less than 50%); or diffuse (positive cells >50%) according to the average density of positive cells for CXCR4 (DAB stained), to see the obvious difference in strength of CXCR4 expression.

### Histomophometry Measurement of Staining Intensity for CXCR4 in Prostate Cancer Tissues

The average density of positive cells (DAB stained) was measured by using Bioquant® Image Analysis Software (RtmBometrics) and an Olympus BX51 Microscope with a Q-Imaging camera. The software analyzed an average group of pixels and returned a data value based on the color value of the pixels in stained samples. Three random fields of prostate tissues were selected at a magnification of (400X) for each section based on the size of the tissue. In each random area, those cells (a group of pixels) that were stained positively (brown) with the CXCR4 antibody were selected by the thresholding tool of the software. The specimen light source is known to affect density measurement; therefore, all sections were measured utilizing the same background correction supplied by Bioquant.

### Subcellular Fractionation

PCa and normal prostate epithelial cells (1×10^6^) were serum-starved for 3 hrs (22RV1 and RWPE1) or 24 hrs (PC3 and DU145), prior to treating with SDF1α (100 ng/ µl) for 30 min. Subcellular fractionations were performed per the manufacturer's instructions (Thermo Scientific). Briefly, cells were lysed in a series of buffers and centrifugation steps to obtain a non-nuclear fraction and an intact nuclear pellet, followed by further lysing to isolate nuclear proteins. Forty to one hundred micrograms of nuclear and non-nuclear fractions were separated by SDS-PAGE electrophoresis and transferred to PVDF membranes. Expression of CXCR4 or GFP-CXCR4 fusion protein was detected with a mouse monoclonal GFP antibody (Santa Cruz; 1∶500) or anti-human CXCR4 antibody (R&D Systems; 1∶1000). Anti-topoisomerase I (Santa Cruz; 1∶1000) and anti-CD44 (Cell Signaling; 1∶1000) antibodies were used to ensure the integrity of fractions and as loading controls. X-ray films were scanned and Quantity One software program was used for densitometry analysis.

### Indirect Immunocytochemistry (ICC) for CXCR4

Cells (3×10^5^) were plated on glass coverslips (Fisher), serum-starved as described, prior to treatments with SDF1α (100 ng/ µl). Cells were fixed with ice-cold 100% methanol for 5 min at −20°C and washed with 1× PBS. Non-specific proteins were blocked in blocking solution (3% normal donkey serum/1% BSA/0.1% Triton X-100 in 1× PBS) for 30 min at RT, prior to incubating with CXCR4 (R&D Systems, 1∶100), Lamin A/C (Santa Cruz, 1∶100), or GFP mouse monoclonal antibody (Santa Cruz, 1∶100) in blocking solution at 4°C overnight. Secondary detection was with Cy3-conjugated donkey anti-mouse IgG or FITC conjugated anti-rabbit IgG (Jackson Immuno Research, 1∶1000) in blocking solution at RT for 1 hr, followed by three washes in 1× PBS. In some cases, nuclei were detected with propidium iodide (1 µg/ µl) or DAPI (1∶250) in 1× PBS prior to mounting in Aqua-Polymount (Polyscience, Inc). Images were taken at Georgia Institute of Technology, Atlanta, GA with a 63x Plan-Apochromat 63x/1.40 Oil DIC objective on a Zeiss LSM-510 UV Confocal Microscope at excitation 488 nm for FITC and 543 nm for Cy3 or at Clark Atlanta University, Atlanta, GA with Axiovision software 4.8.2 on a Zeiss Axio Imager.z1 fluorescence microscope at 40× magnification at excitation 470 nm for FITC, 358 nm for DAPI and 551 nm for Cy3.

### Mutagenesis

R146A and R148A point mutations within the NLS, and deletion of the NLS, within GFP-CXCR4 fusion protein were generated using the Quik Change XL Site-Directed Mutagenesis Kit (Stratagene); pEGFPN1-CXCR4 served as the template [72]. The forward and reverse primers of R146A, R148A and the deleted NLS were (Integrated DNA Technologies): (i) R146A: FWD5′-CACGCCACCAACAGTCAGGCACCAAGGAAGCTGTTGGCTG-3′, REV 5′-CAGCCAACAGCTTCCTTGGTGCCTGACTGTTGGTGGCGTG-3′; (ii) R148A: FWD5`-CCAACAGTCAGAGGCCAGCGAAGCTGTTGGCTGAAA-3`, REV 5`-TTTCAGCCAACAGCTTCGCTGGCCTCTGACTGTTGG-3`; and (iii) NLS deletion: FWD5′-CGCCACCAACAGTCAGCTGTTGGCTGAAAAGG-3′ and REV5′-CCTTTTCAGCCAACAGCTGACTGTTGGTGGCG-3′. The resultant plasmids were pEGFPN1-CXCR4**R146A**, pEGFPN1-CXCR4**R148A** and pEGFPN1-CXCR4**ΔNLS**. Positive CXCR4 mutant clones were selected with kanamycin and further purified by maxi-prep (Omega Bio-tek). Accuracy of the mutations was confirmed by DNA sequencing on an ABI 3130 xl Gene Analyzer Sequencer at Morehouse School of Medicine, Atlanta, GA.

### Transient Transfections

Transient transfections were performed with 2 µg of concentrated DNA and jetPRIME® Polypus transfection, per the manufacturers' instructions. Briefly, PC3 cells were incubated with jetPRIME®-DNA complexes in 15% FBS/RPMI for 4 hrs and the media was replaced with 15% FBS in RPMI for an additional 18 hrs, prior to serum-starvation (24 hrs). Cells were then harvested for respective experiments.

### Expression of Transportinβ1 (TRN1)

Serum-starved cells (5×10^6^) were treated with SDF1α for 30 min prior to harvesting 60 µg of whole cell lysates for western blot analysis. Expression of TRN1 was detected with a mouse monoclonal antibody (Santa Cruz; 1∶1000); α-Tubulin or β-Actin was used as a loading control.

### Immunoprecipitation

One milligram of PC3 whole cell lysates were immunoprecipitated for CXCR4 (Santa Cruz; 1 µg per 250 µg of protein) overnight at 4°C, followed by incubation with Protein A/G Plus-Agarose beads (Santa Cruz) for 2 hrs at 4°C. CXCR4-bound agarose beads were separated from lysate by a series of 3 washes with PBS and centrifugation at maximum speed for 1 min at 4°C. Beads were processed for western blot analysis for TRN1 (Santa Cruz; 1∶1000) and subsequently reprobed for CXCR4 with rabbit anti-CXCR4 (Santa Cruz, 1∶500) antibody followed by incubation with mouse anti-rabbit IgG (Cell Signaling) secondary antibody. Thirty micrograms of the supernatant obtained after incubation with agarose beads were also separated by 10% SDS-PAGE, and processed for western blot analysis for CXCR4 as described in characterization of CXCR4 antibody.

### Short Interfering RNA Transfection

Transient transfection of TRN1 specific siRNA (Santa Cruz) was performed on PC3 cells plated on glass coverslips using JetPRIME®. Briefly, cells (2×10^5^) were plated in 35 mm, 6 well dishes and transfected with 50 nM TRN1-siRNA (Santa Cruz) in 15% FBS/RPMI media at 37°C in 5% CO_2_ for 24 hours. Subsequently, transfected cells were serum-starved for 24 hrs, prior to immunocytochemistry analysis.

### Immunoprecipitation of G_αi_

Serum-starved cells (5×10^6^) were treated with SDF1α for 30 min prior to harvesting for immunoprecipitation. Briefly, cells were washed in 1× PBS and gently scraped in NP-40 lysis buffer (1× PBS pH 7.4, 0.1% Triton × 100, 0.1% NP40 and 1× cocktail inhibitor). After 30 min incubation on ice, the lysate was centrifuged at 600 rcf/5 min/4°C). The supernatant was gently decanted, and the nuclear pellet was resuspended in lysis buffer, 10 times the volume of the nuclei pellet, and sonicated on ice for 3 sec. The lysate was centrifuged at 600 rcf/5 min/4°C, and 1 mg of supernatant was immunoprecipitated for CXCR4 (mouse monoclonal, Santa Cruz) overnight at 4°C, followed by incubation with Protein A/G Plus-Agarose beads (Santa Cruz) for 2 hrs at 4°C. CXCR4-bound agarose beads were separated from lysate by a series of 3 washes with NP40 lysis buffer and centrifugation (5000 pm/2 min/RT). The final wash was with 1× PBS. Beads were processed for western blot analysis and membranes were probed for G_αi_ (Cell Signaling; 1∶1000). Subsequently, the blots were reprobed for CXCR4 with rabbit anti-CXCR4 (Santa Cruz, 1∶500) antibody followed by incubation with mouse anti-rabbit IgG (Cell Signaling) secondary antibody. Topoisomerase1 (Santa Cruz) and anti-CD44 (Cell Signaling) were used to assess the purity of nuclei lysates.

### Intranuclear Calcium (Ca^2+^) Mobilization

Serum-starved PC3 cells (2.5×10^5^) were harvested to obtain intact nuclei in NP-40 lysis buffer as described above, prior to performing assay per the manufacturer's instructions (Enzo Life Sciences). Briefly, untreated isolated nuclei were resuspended in 100 µl of FluoForte dye-loading solution (Enzo Life Sciences) for 45 min at 37°C and 15 min at RT, then centrifuged at 600 rcf/5 min/RT. Solutions of AMD3100 (100 ng/ µl) and pertussis toxin (PTX) (200 ng/ml) were prepared in calcium free, phenol free RPMI. Nuclei samples were resuspended in 100 µl of AMD3100 and PTX, aliquoted into black-walled, clear bottom 96well plates, and incubated for 1 hr. Next, the SDF1α was added to samples in plates, (final dilution 100 ng/ µl) and incubated for 30 min at RT. Intranuclear calcium mobilization was determined by the intensity (increase) of fluorescent (FluoForte)-bound Ca^2+^ in the media. Results were measured on a microplate reader at excitation 490 nm and emission 525 nm. Each sample was prepared in triplicate per experiment, and performed at least three times.

### Statistical Analysis

Where applicable, data were analyzed by a paired student's t-test or ANOVA using GraphPad Prism (GraphPad) software. P values less than 0.05 were considered significant.

## Results

### CXCR4 is Expressed in the Nucleus of Prostate Tissues

**Figure 1:**
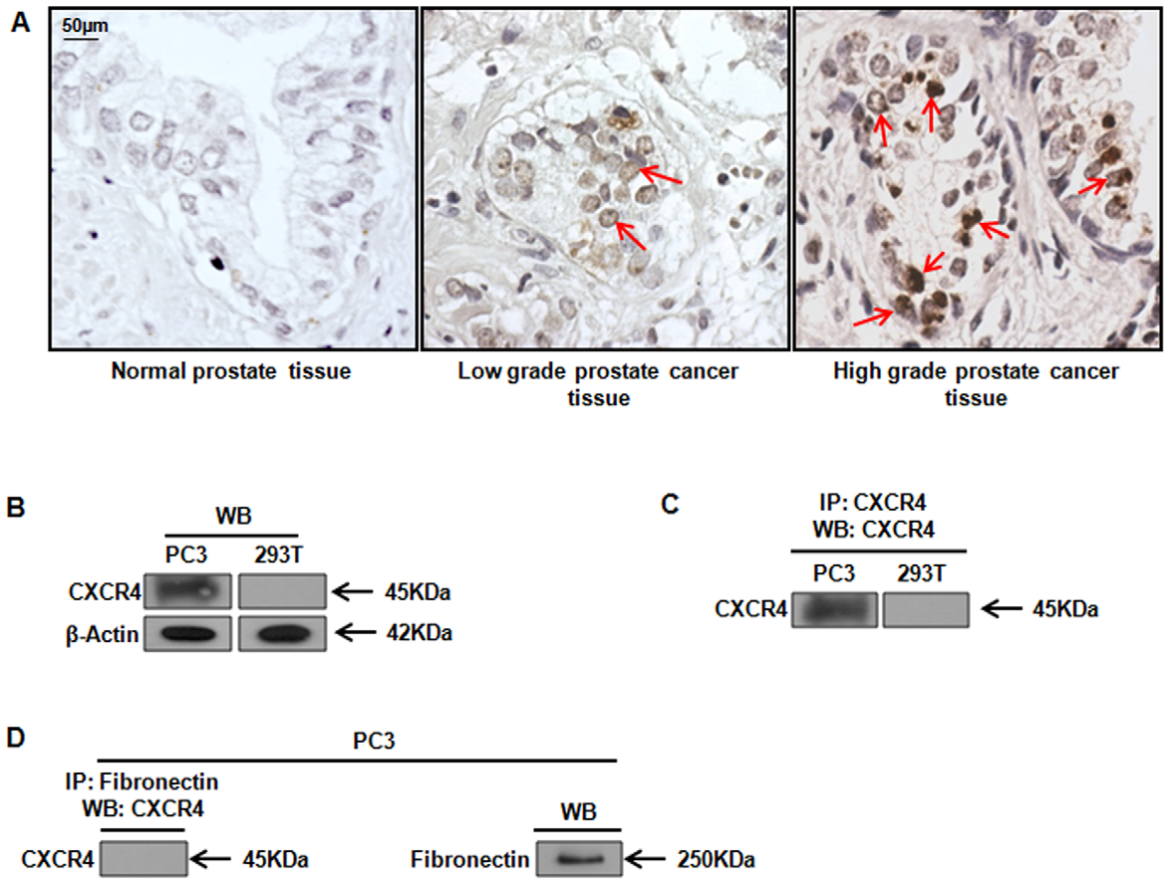
Immunohistochemical (IHC) Staining of Prostate Tissues for CXCR4. ***A***, A human prostate tissue array, ranging from normal to high-grade prostate cancer, was evaluated by IHC for CXCR4 expression using standard methods. Samples were evaluated at magnification 40X, using a Q-Imaging camera of Olympus BX51 Microscope with Bioquant® Image Analysis Software (RtmBometrics). Normal prostate tissues demonstrated slightly weak or undetectable brown staining for CXCR4 (positive cells<5%), and no CXCR4 expression in the nucleus. Representative low grade prostate tissue (grade 2, stage II, T_2_N_0_M_0_, adenocarcinoma) demonstrated random/focal positive staining for CXCR4 in the nucleus (positive cells >11%, but less than 50%), indicating low expression of CXCR4. Representative high grade metastatic prostate tissue (grade 4, stage IV, T_4_N_1_M_1_, adenocarcinoma) demonstrated diffuse/intense staining (positive cells >50%), indicating high expression for CXCR4 in the nucleus. Scale bar represents 50 µm. ***B***, CXCR4 IgG2B mouse monoclonal antibody was evaluated for specificity to CXCR4 protein by western blot analysis in PC3 (CXCR4 positive) or 293T (CXCR4 null) cell lines. ***C***, CXCR4 antibody was evaluated for specificity to CXCR4 protein by immunoprecipitation for CXCR4 and western blot analysis for CXCR4. ***D***, CXCR4 IgG2B antibody was evaluated for specificity to CXCR4 protein by immunoprecipitation with Fibronectin IgG2B mouse monoclonal antibody (unrelated isotype control) and western blot analysis for CXCR4; expression of Fibronectin protein was confirmed by western blot analysis. Beta-actin was used as a loading control.

Previous domain analysis of CXCR4 suggested that CXCR4 contains a nuclear targeting signal between amino acids 90–170 [73]. A bioinformatics analysis using the PSORT II NLS prediction software (http://psort.ims.u-tokyo.ac.jp/) revealed a putative nuclear localization sequence, ‘RPRK’ [72], [74], [75], [76] between amino acids 146–149 within CXCR4 (Table 1). Additionally, a HomoloGene/NCBI database search for the NLS within CXCR4 revealed that ‘RPRK’ is been conserved among species, including chicken, mouse, chimpanzee and others (Table 2). Moreover, CXCR4 has been detected in the nucleus of several cancer tissues [68], [70]. Based on these data, we tested whether CXCR4 protein could be detected within the nucleus of prostate tissues. Using a prostate tissue microarray ranging from normal to high-grade metastatic lesions, we detected positive immunoreactivity for CXCR4 in prostate samples. Positive immunoreactivity was detected as sporadic (CXCR4 positive cells <5%), focal (CXCR4 positive cells >11%, but less than 50%), or diffuse (CXCR4 positive cells >50%), compared to the average total density of positive cells for CXCR4 (DAB stained). Samples with immunohistochemical scores of negative, weak or moderate staining, with sporadic to focal distributions, were considered to have ‘low’ expression, whereas diffuse distributions of staining were considered to have ‘high’ expression for CXCR4. Staining intensity was sporadic to focal in the nucleus of low grade prostate tissues (Fig. 1A), while high grade malignant tissues (Fig. 1A), displayed an increased staining intensity and diffuse expression of CXCR4 throughout the tissue compared to low grade. In both low and high grade tumors, a fraction of CXCR4 clearly co-localized with the nucleus. Staining intensity for CXCR4 was weak, or even null, in normal tissues (Fig. 1A).

**Table 1:** PSORT Prediction of Nuclear Localization Sequence (NLS) in CXCR4.

Gene	Nuclear localization sequence	Amino acid sequence	Position
CXCR4	RPRK	Arg, Pro, Arg, Lys	146 to 149

PSORT is a NLS prediction server to determine the NLS scores of amino acid residues of a protein. It receives amino acid sequence information of a source, e.g., human CXCR4, as inputs and subsequently analyzes the input sequence by applying stored rules for various sequence features of known protein sorting signals. Finally, it reports possible sequences for the input protein to be localized at each candidate site with additional information. The human CXCR4 sequence (NCBI assession number NP_001008540) was searched by the server, which provided a NLS score for each of the 356 residues comprising CXCR4. http://psort.hgc.jp/.

**Table 2:** Multiple Sequence Alignment of the Nuclear Localization Sequence (NLS) Region in CXCR4.

Species	Multiple sequence alignment
Human (NP003458.1)	130ISLDRYLAIVHATNSQRPRKLLAEKVVYVGVWIPALLLTIPDFIFANV177
Mouse (NP034041.2)	132ISLDRYLAIVHATNSQRPRKLLAEKAVYVGVWIPALLLTIPDFIFADVSQ181
Norway rat (NP071541.2)	127ISLDRYLAIVHATNSQRPRKLLAEKAVYVGVWIPALLLTIPDIIFADV174
Dog (NP001041491.1)	131ISLDRYLAIVHATNSQRPRKLLAEKVVYVGVWIPALLLTIPDFIFANV178
Chicken (NP989948.2)	140ISLDRYLAIVHATNSQRPRKLLAEKIVYVGVWLPAVLLTVPDIIFAST187
Chimpanzee (NP001009047.1)	130ISLDRYLAIVHATNSQRPRKLLAEKVVYVGVWIPALLLTIPDFIFANV177

HomoloGene database (NCBI) is a system for automated detection of homologs among the annotated genes of several completely sequenced eukaryotic genomes. Sequences of input organisms are compared then matched into groups using a taxonomic tree built from sequence similarity; highly related organisms are matched up first. http://www.ncbi.nlm.nih.gov/homologene/20739.

We have reported that PC3 cells were positive and 293T cells were null, respectively, for CXCR4 protein [21]. To ensure the specificity of CXCR4 monoclonal antibody (MAB172IgG2b) used to detect its corresponding protein in the nucleus of prostate tissues, we re-analyzed PC3 and 293T for CXCR4 with CXCR4 antibody (MAB172IgG2b) by western blot analysis (Fig. 1B). Similar to our previous studies, CXCR4 was detected in PC3 but not in 293T whole cell lysates (Fig. 1B). Subsequent analysis of cell lysates by immunoprecipitation with MAB172 IgG2b followed by western blot analysis with MAB172 IgG2b detected CXCR4 only in PC3 lysates (Fig. 1C). To further confirm the specificity of MAB172IgG2b to CXCR4, PC3 cell lysates were subjected to immunoprecipitation with Fibronectin IgG2b, an isotype control, followed by western blot analysis with MAB172 IgG2b (Fig. 1D). Fibronectin was not detected by IP with CXCR4, but was detected by western blot with a Fibronectin antibody (Fig. 1D).

### CXCR4 is Present in Nuclear Fractions of Prostate Cancer Cells

**Figure 2:**
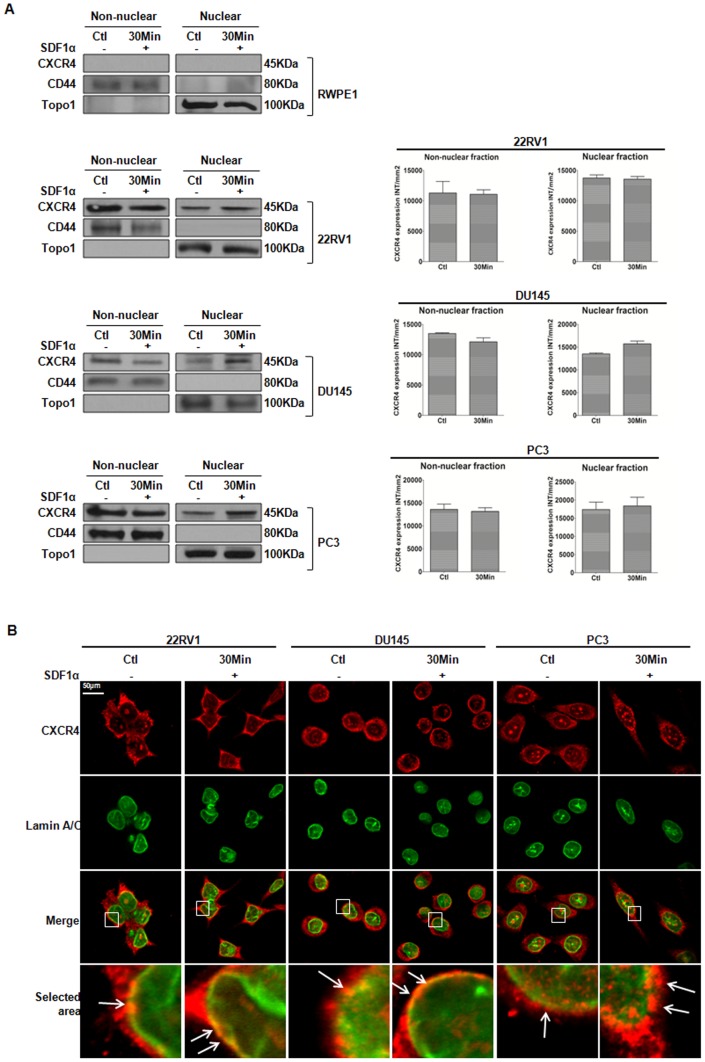
Nuclear CXCR4 Expression in Prostate Cancer Cell Lines. ***A***, Normal prostate epithelial (RWPE1) and PCa (PC3, DU145, 22RV1) cells were stimulated with SDF1α (100 ng/ µl) prior to subcellular fractionation into non-nuclear and nuclear fractions. Immunoblots were probed with anti-CXCR4. Anti-CD44 (non-nuclear) and anti-Topoisomerase1 (Topo 1, nuclear) were used as markers for fractionation purity and as loading controls. The bar graphs are quantitative results of the band density representing expression of CXCR4 in each fraction. Data were mean +S.E. from three independent experiments. *, P<0.05. ***B***, Immunocytochemistry of PCa cell lines for CXCR4. PCa cells were stimulated with SDF1α (100 ng/ µl), fixed with methanol, blocked then incubated with an antibody mixture containing a mouse anti-CXCR4 monoclonal antibody and a rabbit polyclonal anti-Lamin A/C antibody, followed by secondary mixture containing a Cy3-conjugated anti-mouse antibody and FITC-conjugated anti-rabbit antibody. Imaging was with a Zeiss LSM-510 UV Confocal Microscope using the 63× Plan-Apochromat 63x/1.40 Oil DIC objective at excitation 488 nm for FITC and 543 nm for Cy3. Confocal images demonstrating the plasma membrane and cytosolic localization of CXCR4 (red), intact nuclear membrane (green), and nuclear-associated localization of CXCR4 (yellow/orange) are shown. Small arrows indicate co-localization of CXCR4 with the nucleus (yellow/orange). Scale bars represent 50 µm.

We used biochemical fractionation to confirm the nuclear localization of CXCR4 detected in our tissue staining. PCa cell lines were fractionated into nuclear and non-nuclear samples for detection of CXCR4 by western blot analysis (Fig. 2A). We found that normal prostate epithelial cells (RWPE1) were null for CXCR4 [77]; however, three CXCR4-expressing PCa cell lines (22RV1, DU145 and PC3) dually expressed CXCR4 in both nuclear and non-nuclear fractions independent of SDF1α stimulation (Fig. 2A). The purity of subcellular fractions was confirmed by expression of CD44, a non-nuclear marker [78], and topoisomerase 1, a nuclear marker [79], which ruled out the possibility that expression of CXCR4 observed in nuclear fractions was due to contamination.

We further confirmed CXCR4 in nuclei of PCa cell lines by indirect immunohistochemistry (ICC) (Fig. 2B). In all three cell lines, we found CXCR4 to be ubiquitously expressed throughout the cells, or as distinct foci around the nucleus, at the PM and in the cytoplasm, independent of SDF1α treatment. Collectively, these observations suggest that CXCR4 is expressed at the PM, but also associated with the nucleus of PCa cells.

### A Putative NLS in CXCR4

**Figure 3:**
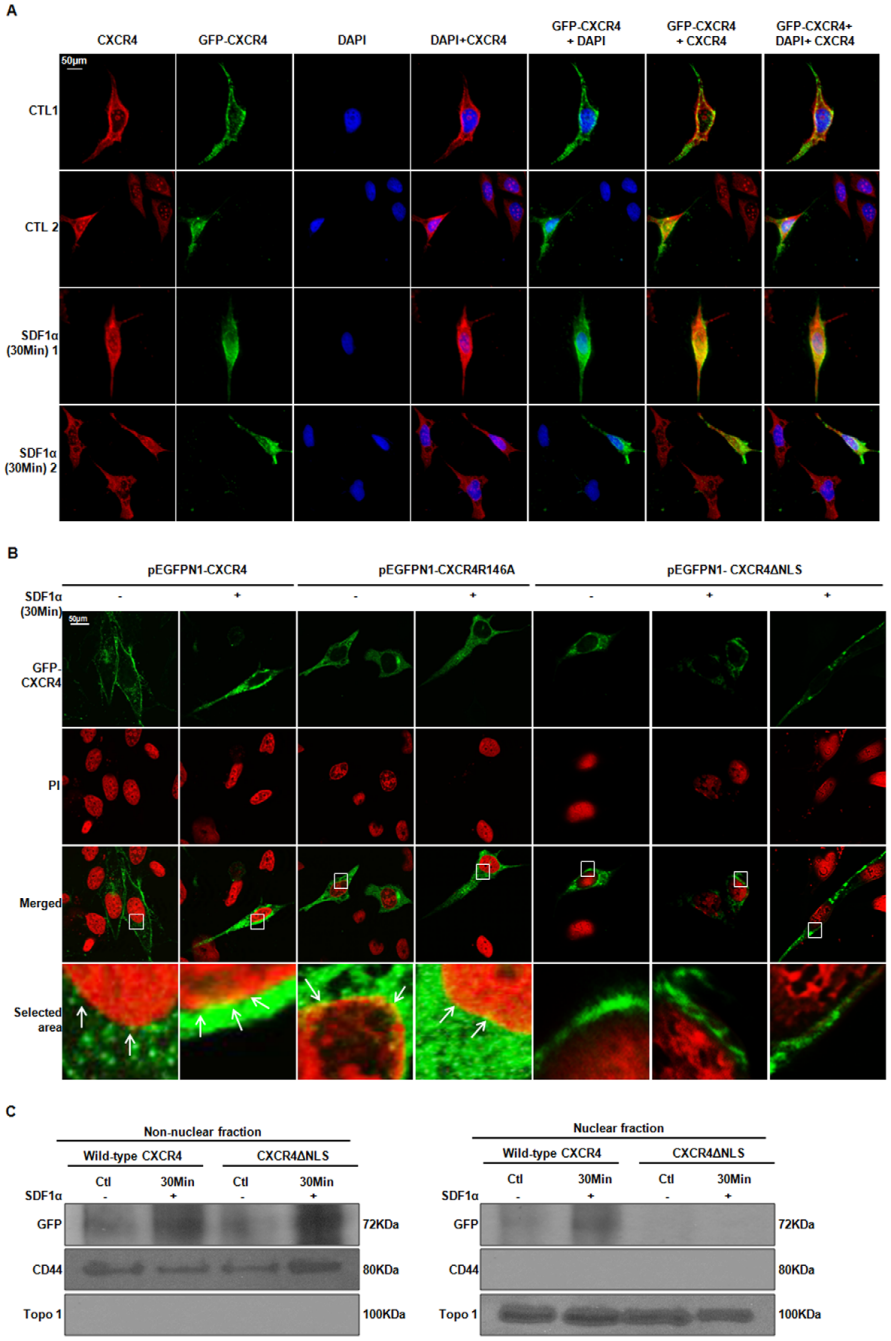
A Putative Functional NLS within CXCR4. ***A***, GFP-CXCR4 fusion protein localized similar to endogenous CXCR4. CXCR4-pEGFPN1 transfected PC3 cells were stimulated with SDF1α, fixed with methanol, blocked then incubated with a mouse anti-CXCR4 monoclonal antibody, followed by a Cy3-conjugated anti-mouse secondary antibody. Nuclei were stained with DAPI (blue). Images were taken at 40× maginification using Axiovision software 4.8.2 with a Zeiss Axio Imager.z1 fluorescence microscope at ex = 470 nm for FITC, ex = 358 nm for DAPI and ex = 551 nm for Cy3. Images demonstrate the co-localization (yellow) of endogenous CXCR4 (red) with GFP-tagged CXCR4 (green). ***B***, Localization analysis of wild type CXCR4 (CXCR4-pEGFPN1), NLS-mutant of CXCR4 (pEGFPN1-CXCR4**R146A**,) and deleted NLS of CXCR4 (CXCR4**ΔNLS**) by immunocytochemistry in PC3 cells. Nuclei were stained with propidium iodide (red) and CXCR4 was detected as the fusion protein GFP-CXCR4 (green). Imaging was with a Zeiss LSM-510 UV Confocal Microscope using the 63× Plan-Apochromat 63x/1.40 Oil DIC objective at ex = 488 nm for FITC and ex = 543 nm for Cy3. Scale bars represent 50 µm. ***C***, Transfected cells were stimulated with SDF1α prior to subcellular fractionation into non-nuclear and nuclear fractions. Immunoblots were probed with anti-GFP to detect the fusion protein GFP-CXCR4. Anti-CD44 (non-nuclear) and anti-Topoisomerase1 (Topo 1, nuclear) were used as markers for fractionation purity and as loading controls.

Nuclear-localized PM receptors must contain a nuclear localization sequence (NLS) to permit transport to and/or into the nucleus [80]. To determine whether the PSORT II-predicted NLS, ‘^146^RPRK^149^’ was functional and contributed to nuclear expression of CXCR4, we evaluated the intracellular distribution of wildtype GFP-tagged CXCR4 (pEGFPN1-CXCR4, GFP-CXCR4 fusion protein), two mutated fusion proteins in which arginine 146 and 148 were separately mutated to an alanine (CXCR4**R146A** and CXCR4**R148A**, respectively), as well as a fusion protein where the NLS was deleted (CXCR4**ΔNLS**). Plasmids encoding GFP-CXCR4 were transfected into PC3 cells and examined by ICC microscopy (Fig. 3). The localization pattern of GFP-CXCR4 at the plasma membrane and in the cytoplasm was consistent with endogeneous CXCR4 (Fig. 3A). Previous studies have reported an expression pattern for GFP-CXCR4 similar to our observation in other cancer cell lines [81], [82]. Wild-type GFP-tagged CXCR4 was localized predominantly at the PM, with some localization at the nucleus in untreated cells (Fig. 3B). However, an increase in punctate staining was observed at the nucleus/nuclear membrane upon treatment with SDF1α. Interestingly, both CXCR4**R146A** (Fig. 3B) and CXCR4**R148A** (data not shown) were detectable at the nucleus, suggesting that neither **R146A** nor **R148A** mutations in the NLS were sufficient to inhibit CXCR4 localization to the nucleus. To further examine the requirement of this NLS to localize CXCR4 to the nucleus, we deleted the NLS, ‘^146^RPRK^149^’, within pEGFPN1-CXCR4 (CXCR4**ΔNLS**). We detected CXCR4**ΔNLS** at the PM and diffusely throughout the cytosol, similar to wild-type GFP-CXCR4, but we did not detect CXCR4ΔNLS at the nucleus (Fig. 3B). To further confirm that CXCR4**ΔNLS** was excluded from the nucleus, PC3 cells were transiently transfected with wildtype GFP-CXCR4 or CXCR4**ΔNLS** then fractionated into nuclear and non-nuclear samples for analysis by western blot analysis (Fig. 3C). Consistent with ICC observations, we found that wild type GFP-CXCR4 and CXCR4**ΔNLS** were both detectable in non-nuclear fractions, while only GFP-CXCR4 was detected in nuclear fractions. Collectively, these data suggest that the ‘RPRK’ motif may be involved in localization of CXCR4 to the nucleus in prostate cancer.

### CXCR4 Demonstrated an Interaction with Transportinβ1

**Figure 4:**
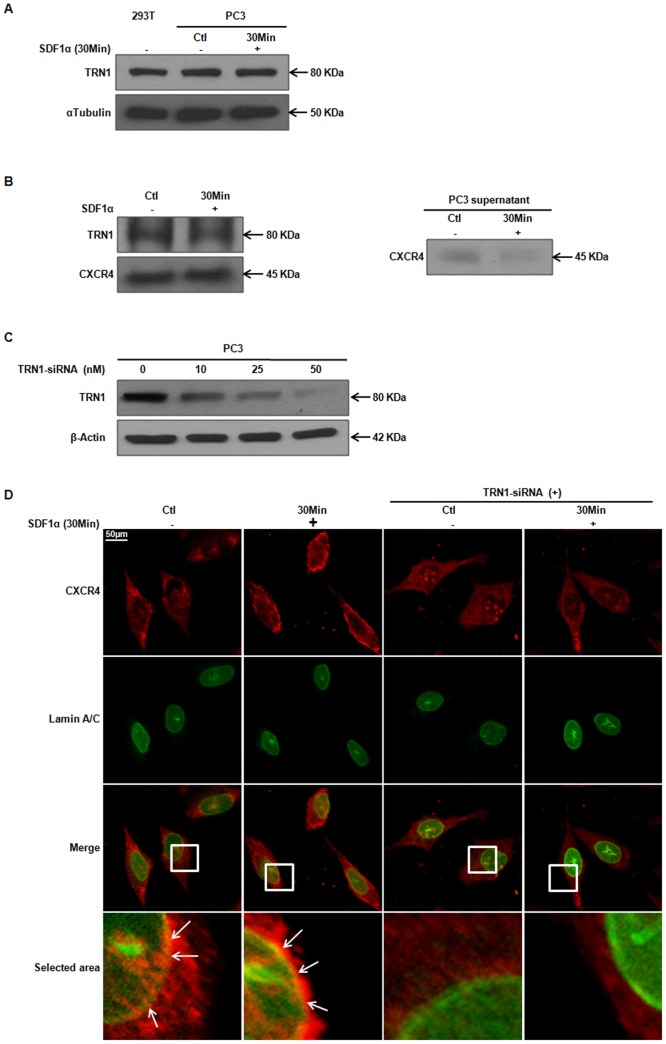
CXCR4 and TRN1 Demonstrate an Interaction. ***A***, Sixty micrograms of total protein were analyzed for TRN1 expression by western blot analysis using a TRN1 specific antibody. Alpha-tubulin served as a loading control. ***B***, One milligram of PC3 whole cell lysate was immunoprecipitated with anti-CXCR4 and separated by SDS-PAGE. Immunocomplexes were probed with anti-TRN1 or anti-CXCR4 to ensure that CXCR4 interacted with TRN1 and was immunoprecipitated, respectively. Thirty micrograms of whole cell PC3 supernatant, post-immunoprecipitation, were separated by SDS-PAGE and harvested for western blot analysis to assess the efficiency of CXCR4 immunoprecipitation. ***C and D***, Cells were transiently transfected with TRN1-specific siRNA to determine an effective concentration *(* ***C*** *)*, prior to harvesting for immunohistochemistry with anti-Lamin A/C and anti-CXCR4 *(* ***D*** *)*. Images were taken using Zeiss Axio Imager.z1 fluorescence microscope at 40× magnification at excitation 470 nm for FITC and 551 nm for Cy3. Small arrows indicate co-localization of CXCR4 with the nucleus (yellow/orange). Scale bar represents 50 µm.

We identified a putative NLS motif that could be critical for CXCR4 nuclear localization; however, the motif ‘RPRK’ is not a typical classical NLS [60]. In fact, such sequences can also mediate direct binding to other transport receptors. Among the different molecules that are involved in the transport of various cargos to the nucleus, members of the karyopherin beta (β) family contribute directly or indirectly to the nuclear shuttling of molecules [83]. Transportinβ1 (TRN1), also known as Karyopherinβ2, is a transport molecule of the importin-β family that has been linked to desensitization [63] and nuclear-cytoplasmic shuttling of receptors [84], [85], [86], [87], [88], [89]. To test whether TRN1 was involved in CXCR4 transport to the nucleus, we first established that PC3 cells expressed TRN1 by western blot analysis (Fig. 4A); 293T cells served as a positive control for TRN1 expression. Next, we tested for an interaction between CXCR4 and TRN1. We immunoprecipitated CXCR4 from whole cell lysates and tested for co-purification of TRN1 by western blot analysis. Figure 4B demonstrates that CXCR4 and TRN1 associated in PC3 cell lysates. To test whether the association between CXCR4 and TRN1 led to CXCR4 translocation to the nucleus, we decreased TRN1 protein expression by siRNA (Fig. 4C), then determined CXCR4 localization by ICC. We first confirmed an effective siRNA-mediated depletion of TRN1 by western blot analysis (Fig. 4C). As previously described in control cells, CXCR4 localized to the PM, in the cytoplasm and in distinct foci around the nucleus. When TRN1 expression was diminished, CXCR4 was undetectable around the nucleus, even in the presence of SDF1α (Fig. 4D).

### Nuclear-associated CXCR4 is Functional

**Figure 5:**
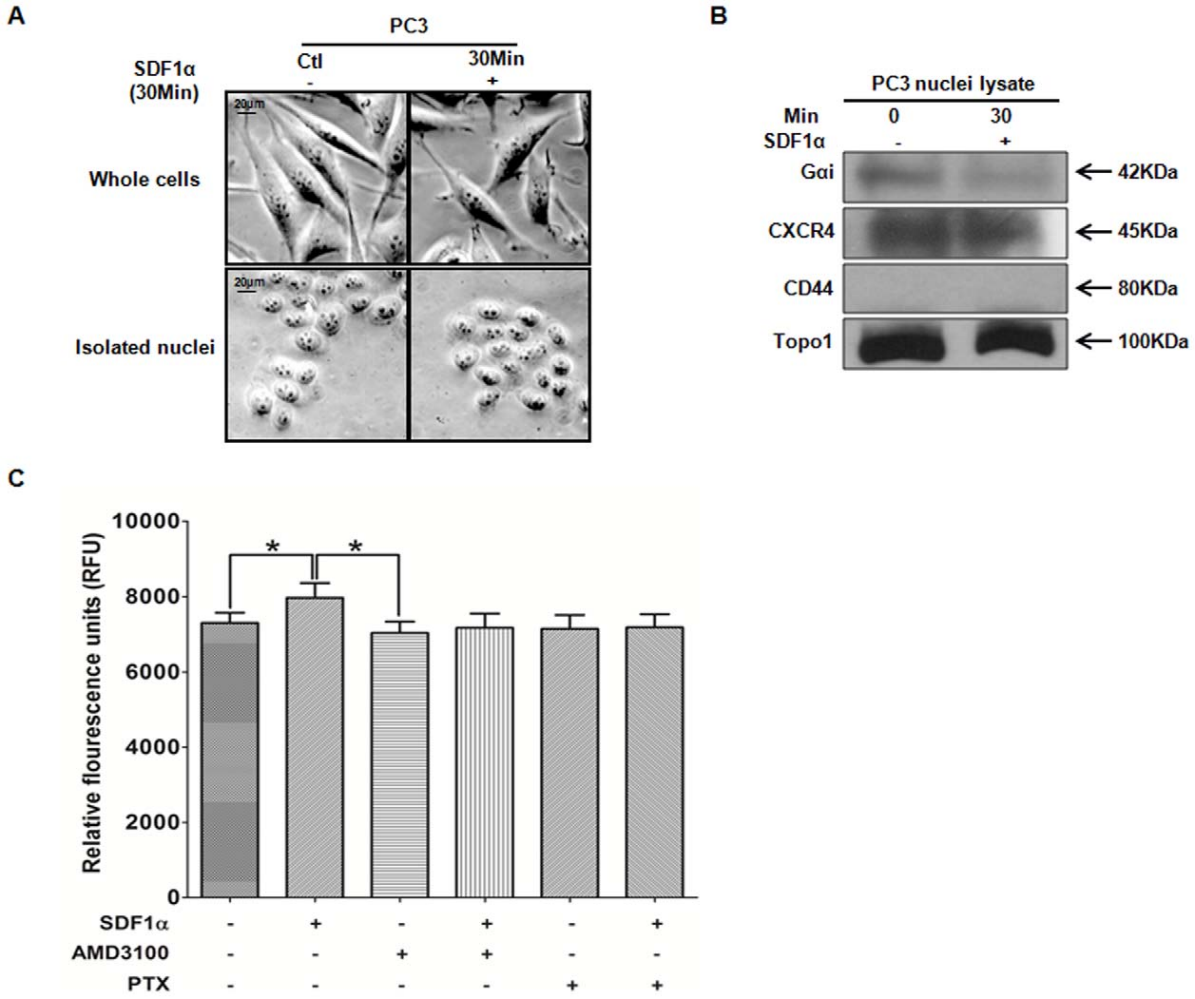
Nuclear CXCR4 was Functional at the Nucleus. ***A***, Representative light images of whole cells and isolated nuclei confirmed the integrity of nuclear isolation at 20× magnification. ***B***, Whole cells were treated with SDF1α prior to isolating and lysing intact nuclei. Nuclei lysates (1 mg) were immunoprecipitated with anti-CXCR4 and separated by SDS-PAGE. Immunocomplexes were probed for G_αi_ (first row) or CXCR4 antibody (second row), respectively. Anti-CD44 (non-nuclear) and anti-Topoisomerase1 (Topo1, nuclear) were used as markers for fractionation purity and as loading controls. ***C***, PC3 nuclei were isolated, incubated with FluoForte dye Ca^2+^ probe, followed by incubation with AMD3100 or pertussis toxin (PTX) for 1 hr, then stimulated with SDF1α for 30 min. An increase in fluorescent-bound Ca^2+^ was measured on a microplate reader at ex = 490 nm/em = 525 nm.

Finally, we sought to determine whether CXCR4 receptors at the nucleus are functional. Upon ligand stimulation and activation, GPCRs, including CXCR4, dissociates from a trimer of G-proteins (G_α_ and G_βγ_) which initiates secondary signaling pathways, such as increased cyclic AMP, increased intracellular calcium levels, and others [90]. Thus, a reduction in the G-alpha protein, G_αi_, associated with CXCR4 represents an active state of a receptor. Therefore, we co-immunoprecipitated CXCR4 and G_αi_ and determined G_αi_ expression levels by Western blot analysis, to assess whether nuclear-associated CXCR4 was active. Whole cells were stimulated with SDF1α then harvested to isolate intact nuclei (Fig. 5A). Nuclei were lysed, then immunoprecipitated with anti-CXCR4, prior to immunobloting for associated G_αi_. In untreated cells, we observed a basal level in G_αi_ expression, which decreased upon treatment with SDF1α, suggesting that nuclear-associated CXCR4 is functional and can respond to SDF1α.

We further tested the functionality of nuclear-associated CXCR4 by assessing whether the receptor stimulated release of intra-nuclear Ca^2+^. Isolated intact PC3 nuclei were incubated with Ca^2+^-binding fluorescent probe (FluoForte® dye), then incubated with CXCR4 antagonist, AMD3100, or G_αi_/G_αo_ inhibitor pertussis toxin (PTX), separately for one hour, followed by treating with SDF1α. Calcium mobilization was determined by an increase in fluorescent probe in the media, and measured at ex = 490 nm/em = 525 nm. We observed a significant increase in intra-nuclear Ca^2+^ release from nuclei stimulated with SDF1α, compared to untreated samples. Further, AMD3100 antagonized CXCR4 function, which resulted in decreased Ca^2+^ levels, lower than SDF1α-treated samples. PTX prevents G-proteins from interacting with GPCRs, thus interfering with intracellular communication. As such, we did not observe an increase in Ca^2+^-mobilization in PTX-treated nuclei, even in the presence of SDF1α, supporting that the intra-nuclear Ca^2+^ surge was evoked by CXCR4, yet sensitive to PTX inhibition. Our results are in agreement with earlier studies that observed functional GPCRs associated with the nucleus, and that these nuclear GPCRs mobilized Ca^2+^ [40], [91], [92].

## Discussion

This study established that CXCR4 receptor protein is expressed in PCa cells and is associated with the nucleus in these cells. Additionally, we provide insight into a specific nuclear protein import mechanism that contributes to nuclear localization of CXCR4. Importantly, CXCR4 responded to its ligand, SDF1α, at the nucleus. Nuclear localization of CXCR4 may be a mechanism by which prostate cancer cells employ to survive, even after the insult of chemotherapy. Therefore, antagonizing nuclear transport pathways and/or the action of nuclear CXCR4, could provide a rational approach to prevention and management of prostate cancer.

PCa mortality is often a result of metastasis to secondary organs. CXCR4 is involved in the metastatic spread of primary tumor cells through activation of requisite pathways, which signals to downstream targets for invasion and movement through the vasculature, the establishment of a blood supply at the new tumor site and inhibition of immunosurveillance mechanisms that will destroy the new tumor [22]. Taichman *et al*. [23] initially observed that CXCR4 facilitated PCa metastasis to the bone, the primary site of distal PCa colonization. SDF1α was constitutively expressed in the bone marrow by osetoblasts, fibroblasts, and endothelial cells, which directed cell migration, by attracting PCa cells that expressed CXCR4 on the plasma membrane. To date, CXCR4, like all GPCRs, is regarded as a plasma membrane receptor. However, an emerging concept is that GPCRs are translocated to the nucleus and other intracellular organelles, possibly after internalization [93]. For instance, Wright *et al*. reported that endogenous α1-adrenergic receptors (α1-ARs) localized to and signaled at nuclei in adult cardiac myocytes [94], and in 2012, the group described that α1-AR nuclear localization drove the formation of receptor oligomers and regulated signaling in adult cardiac myocytes [95], suggesting that GPCRs at the nucleus exhibited the same behavior as their PM counterparts.

The biochemical mechanisms of CXCR4 intracellular localization to the nucleus, and subsequent functions, are not well understood. The generally accepted view of CXCR4 localization and signaling centers upon a PM-localized receptor that is activated by an extracellular ligand, SDF1α. This ligand then initiates intracellular signals through G-proteins that support favorable responses for tumor development. Following activation and subsequent signaling, the canon concept of events is that CXCR4 is rapidly internalized from the PM to attenuate signaling communication, and then recycled back to the PM as an inactivated receptor, or sorted to the lysosome for degradation; both processes are important for receptor signal termination [96], [97], [98]. Consistent with our findings reported here, GPCRs that have been detected at the nucleus were reported to have originated from the PM [38], [40], [99], [100].

The results of this study support an alternately-localized and functional CXCR4 receptor. As assessed by decreased G_αi_ expression and Ca^2+^ mobilization in the presence of SDF1α, CXCR4 participates in intra-nuclear signaling at the nucleus. Nuclear CXCR4 was initially observed in diverse tumor tissues [69], [70], [101], [102], [103] and correlated with significant predictors for poor overall malignant survival [103], lymphovascular invasion [104] and lymph node metastasis [105]. Our findings extended these observations further and provide evidence that: (i) CXCR4 was present at the nucleus of prostate tumor tissues and cell lines; (ii) nuclear CXCR4 contained a putative, functional NLS which excluded CXCR4 from the nucleus when deleted; and (iii) CXCR4 associated with TRN1 and depletion of TRN1 decreased localization of CXCR4 to the nucleus.

To date, considerable evidence supports the presence of GPCRs at the PM, or within the perinuclear/nuclear compartments of cells, following ligand activation [40], [99], [106], [107]. It is well known that CXCR4 is highly expressed in malignant PCa cells [108] and is involved in metastasis [21], [22], [24], [109]; however, few reports have observed a subcellular localization for CXCR4 other than the PM and endosomes in tumor tissues. We observed positive staining for CXCR4 in the nucleus of PCa tissues; furthermore, the amount of nuclei positively stained for CXCR4 increased with the grade of the tumor. In PCa cell lines, unexpectedly, we detected CXCR4 in nuclear fractions of untreated cells; nuclear CXCR4 expression increased with SDF1α stimulation. Sun *et al*. demonstrated that PCa cells expressed SDF1α mRNA and secreted a biologically active protein [110]. Perhaps the nuclear expression of CXCR4 in untreated cells may result from autocrine signaling [111], [112]. Endogenous SDF1α ligand secreted by PCa cells may act upon PM-localized CXCR4 in an autocrine manner, resulting in receptor internalization and subsequent nuclear targeting of CXCR4. Our results do not support a correlation between increased nuclear CXCR4 expression and PCa metastasis, or the clinical relevance of nuclear CXCR4 expression in predicting PCa prognosis survival. However, Woo *et al*. predicted that high expression of nuclear CXCR4 in hormone receptor negative breast cancer was associated with the high possibility of lymph node metastasis [113].

The origin and functional relevance of nuclear CXCR4, and the precise mechanisms involved in its nuclear translocation, have yet to be established. Wang *et al*. presumed the presence of a non-traditional, nuclear localization sequence, ‘^146^RPRK^149^’, within CXCR4, using C-terminal deletant plasmid constructs to reveal expression of CXCR4 at the nucleus [73]. Nuclear localization sequences are a stretch of positively charged, highly basic amino acids (lysines or arginines) [114] and are divided into two types: (i) the classical monopartite type containing a single cluster of 4–6 lysine(K)/arginine (R) amino acid residues; and the (ii) the bipartite type contains two clusters of basic amino acid residues separated by 10–12 amino acids [115]. The majority of non-classical types of NLSs consist of a single 20–40 long stretch of non-basic amino acids [116], [117], [118]. Some non-classical NLSs do not follow these rules, as they consist of a single short stretch of one or more basic amino acids that are distinct from the monopartite NLS [66], [119], [120], [121], [122], such as the motif ‘RPRK’. Ren *et al*. reported that mammalian target of rapamycin (mTOR) contained ‘RPRK’, and upon deletion, onion cells loss nuclear expression of mTOR [75].

Transportinβ1 studies on plasma membrane GPCRs have reported that they are functional and able to initiate signaling at the nucleus [38], [40], [91], [99]. Of particular interest, the importins beta, to which TRN1 belongs, were involved in nuclear localization of GPCRs, such as angiotensin 1, opiod growth factor, fibroblast growth factor receptors and CCR2 chemokine receptor [57], [84], [88], [123], [124], [125]. The canon concept of GPCR signaling is that signal transduction cascades are initiated at the PM, but not at the nuclear membrane. However, this concept has been disapproved by evidence which demonstrated that the nuclear envelope plays a major role in signaling cascades. For instance, GPCR-associated heterotrimeric G proteins [126], and downstream signaling molecules, such as adenylate cyclase [127], phospholipase C [128] and phospholipase D [126], have been found localized at the nucleus. Moreover, nuclear membranes have been shown to possess signaling molecules such as 1,4,5-triphosphate and inositol 1,3,4,5-tetrakisphosphate [128], further confirming the role that nuclear membranes play in signal transduction. We observed that nuclear CXCR4 was associated with G_αi_ in untreated samples; G_αi_ dissociated from CXCR4 upon SDF1α stimulation, compared to untreated samples, further confirming that nuclear CXCR4 is functional. Most studies that have reported functional nuclear GPCRs observed an increase in intra-nuclear Ca^2+^ levels upon stimulation with an appropriate agonist [40], [45], [91], emphasizing an importance of nuclear GPCRs to cells, since nuclear Ca^2+^ play pivotal roles in nuclear functions (cell division, proliferation, protein import, apoptosis, and gene transcription) [129]. Among the various reports where nuclear calcium was mobilized from organelles, such as the cytoplasm [130], nuclear lumen [131] and nucleoplasmic reticulum [132], the latter two have been suggested to increase/enhance signals initiated in the cytoplasm, and/or generate its own Ca^2+^ transients [131], [133]. Additionally, the amplitude and duration of calcium signals have also been shown to differentially control activation of transcription factors [134]. For instance, transcription factors, such as NF-κB, c-Jun, and N-terminal kinase are activated by transient increases in Ca^2+^ [134]. Taken together, data herein, and by our colleagues, emphasize a secondary GPCR signaling network at the nucleus, which may ensure, or enhance, the communication and coordination of numerous biochemical signals that are critical in regulating tumorigenic paradigms within the cell.

In conclusion, the data presented provides the first clear evidence of CXCR4 located at the nucleus of cancer cells and existing as a functional, ligand-responsive receptor in advanced metastatic PCa cells. Therefore, antagonizing the action of nuclear CXCR4 could provide a rational approach to the prevention and management of PCa metastasis. Lee *et al*. [135] described a unique survival system in breast cancer cells by which VEGF acted as an intracrine survival factor through its binding to nuclear VEGFR1. This theory may hold true for tumor cells that express CXCR4 at the nucleus, and secrete SDF1α from the same cell. This study is significant to therapeutic development, as a functional CXCR4 receptor that can initiate signaling from inside the cell may escape from chemotherapeutic agents that are designed to antagonize the PM receptor, and/or cannot pass through the hydrophobic regions of the PM to reach nuclear receptors.

## References

[1] Jemal A, Siegel R, Ward E, Hao Y, Xu J, et al (2009) Cancer statistics 2009. CA Cancer J Clin 59: 225–249. 19474385 10.3322/caac.20006

[2] Sneyd M (2008) Ethnic differences in prostate cancer survival in New Zealand: a national study. Cancer Causes Control 19: 993–999. 18478341 10.1007/s10552-008-9166-1

[3] Cancer IN (2009) Surveillance Research Program, Cancer Statistics Branch. Surveillance, Epidemiology, and End Results (SEER) Program Populations (1969–2007). November 2009 edition.

[4] Vindrieux D, Escobar P, Lazennec G (2009) Emerging roles of chemokines in prostate cancer. Endocrine-Related Cancer 16: 663–673. 19556286 10.1677/ERC-09-0109

[5] Rollins B (1997) Chemokines. Blood 90: 909. 9242519

[6] Premack B, Schall TJ (1996) Chemokine receptors: gateways to inflammation and infection. Nat Med 2: 1174. 8898734 10.1038/nm1196-1174

[7] Luster A (1998) Chemokines–chemotactic cytokines that mediate inflammation. N Engl J Med 338: 436. 9459648 10.1056/NEJM199802123380706

[8] Palczewski K (2006) G Protein–Coupled Receptor Rhodopsin. Annu Rev Biochem 75: 743–767. 16756510 10.1146/annurev.biochem.75.103004.142743 PMC1560097

[9] Patel T (2004) Single Transmembrane Spanning Heterotrimeric G Protein-Coupled Receptors and Their Signaling Cascades. Pharmacol Rev 56: 371–385. 15317909 10.1124/pr.56.3.4

[10] Neer E (1995) Heterotrimeric G proteins: Organizers of transmembrane signals. Cell Cycle 80: 249–257. 10.1016/0092-8674(95)90407-7 7834744

[11] Gautam N, Downes G, Yan K, Kisselev O (1998) The G-protein βg complex Cell Signal. 10: 447–455. 10.1016/s0898-6568(98)00006-0 9754712

[12] Lefkowitz R (1993) G protein-coupled receptor kinases.. Cell Cycle 74: 409–412. 10.1016/0092-8674(93)80042-d 8394218

[13] Ferguson S, Barak L, Zhang J, Caron M (1996a) Can G-protein-coupled receptor regulation: Role of G-protein-coupled receptor kinases and arrestins. J Physiol Pharmacol 74: 1095–1110. 10.1139/cjpp-74-10-1095 9022829

[14] Ferguson S, Caron MG (1998) G protein-coupled receptor adaptation mechanisms. Semin Cell Dev Biol 9: 119–127. 9599406 10.1006/scdb.1997.0216

[15] Krupnick J, Benovic J (1998) The role of receptor kinases and arrestins in G protein-coupled receptor regulation.. Annu Rev Pharmacol Toxicol 38: 289–319. 9597157 10.1146/annurev.pharmtox.38.1.289

[16] Hall R, Premont R, Lefkowitz R (1999) Heptahelical receptor signaling:Beyond the G protein paradigm. J Cell Biol 145: 927–932. 10352011 10.1083/jcb.145.5.927 PMC2133121

[17] Luttrell L, Daaka Y, Lefkowitz R (1999a) Regulation of tyrosine kinase cascades by G-protein-coupled receptors. Curr Opin Cell Biol 11: 177–183. 10209148 10.1016/s0955-0674(99)80023-4

[18] Schoneberg T, Schultz G, Gudermann T (1999) Structural basis of G proteincoupled receptor function.. Mol Cell Endocrinol 151: 181–193. 10411333 10.1016/s0303-7207(99)00017-9

[19] Muller A, Homey B, Soto H, Ge N, Catron D, et al (2001) Involvement of chemokine receptors in breast cancer metastasis. Nature 410: 50–56. 11242036 10.1038/35065016

[20] Akashi T, Koizumi K, Tsuneyama K, Saiki I, Takano Y, et al (2008) Blackwell Publishing Asia Chemokine receptor CXCR4 expression and prognosis in patients with metastatic prostate cancer. Cancer Sci 99: 539–542. 18201276 10.1111/j.1349-7006.2007.00712.x PMC11158982

[21] Chetram M, Odero-marah V, Hinton C (2011) Loss of PTEN Permits CXCR4-Mediated Tumorigenes is through ERK1/2 in Prostate Cancer Cells. Molecular Cancer Research 9: 89–102. 10.1158/1541-7786.MCR-10-0235 PMC3443870 21076047

[22] Chetram M, Don-Salu-Hewage A, Hinton C (2011) ROS enhances CXCR4-mediated functions through inactivation of PTEN in prostate cancer cells. Biochem Biophys Res Commun 410: 195–200. 21627959 10.1016/j.bbrc.2011.05.074 PMC3163383

[23] Taichman R, Cooper C, Keller E (2002) Use of the Stromal Cell-derived Factor-1/CXCR4 Pathway in Prostate Cancer Metastasis to Bone. Cancer Res 62: 1832–1837. 11912162

[24] Darash-Yahana M, Pikarsky E, Abramovitch R, Zeira E, Pal B, et al (2004) Role of high expression levels of CXCR4 in tumor growth, vascularization, and metastasis. FASEB J 18: 1240–1242. 15180966 10.1096/fj.03-0935fje

[25] Kozin S, Kamoun W, Huang Y, Dawson M, Jain R, et al (2010) Recruitment of myeloid but not endothelial precursor cells facilitates tumor regrowth after local irradiation. Cancer Res 70: 5679–5685. 20631066 10.1158/0008-5472.CAN-09-4446 PMC2918387

[26] Rubin J, Kung A, Klein R (2003) A small-molecule antagonist of CXCR4 inhibits intracranial growth of primary brain tumors. Proc Natl Acad Sci U S A 100: 13513–13518. 14595012 10.1073/pnas.2235846100 PMC263845

[27] Bleul CC, Farzan M, Choe H, Parolin C, Clark-Lewis I, et al (1996) The lymphocyte chemoattractant SDF-1 is a ligand for LESTR/fusin and blocks HIV-1 entry. Nature 382: 829–833. 8752280 10.1038/382829a0

[28] Chinni SR, Sivalogan S, Dong Z, Filho JC, Deng X, et al (2006) CXCL12/CXCR4 signaling activates Akt-1 and MMP-9 expression in prostate cancer cells: the role of bone microenvironment-associated CXCL12. Prostate 66: 32–48. 16114056 10.1002/pros.20318

[29] Chinni SR, Yamamoto H, Dong Z, Sabbota A, Bonfil RD, et al (2008) CXCL12/CXCR4 transactivates HER2 in lipid rafts of prostate cancer cells and promotes growth of metastatic deposits in bone. Mol Cancer Res 6: 446–457. 18337451 10.1158/1541-7786.MCR-07-0117 PMC3842603

[30] Kukreja P, Abdel-Mageed AB, Mondal D, Liu K, Agrawal KC (2005) Up-regulation of CXCR4 expression in PC-3 cells by stromal-derived factor-1alpha (CXCL12) increases endothelial adhesion and transendothelial migration: role of MEK/ERK signaling pathway-dependent NF-kappaB activation. Cancer Res 65: 9891–9898. 16267013 10.1158/0008-5472.CAN-05-1293

[31] Gilman A (1987) G proteins: Transducers of receptor-generated signals. Annu Rev Biochem 56: 615–649. 3113327 10.1146/annurev.bi.56.070187.003151

[32] Stow J, de Almeida JB, Narula N, Holtzman E, Ercolani L, et al (1991) A heterotrimeric G protein on Golgi membranes regulates the secretion of a heparan sulfate proteoglycan in LLC-PK1 epithelial cells. J Cell Biol 114: 1113–1124. 1910049 10.1083/jcb.114.6.1113 PMC2289129

[33] Audigier Y, Nigam S, Blobel G (1998) Identification of a G protein in rough endoplasmic reticulum of canine pancreas. J Biol Chem 263 16 352–357. 3141406

[34] Carlson K, Woolkalis M, Newhouse M, Manning D (1986) Fractionation of the beta subunit common to guanine nucleotide binding regulatory proteins with the cytoskeleton. Mol Pharmacol 30: 463–468. 3095628

[35] Crouch M (1991) Growth factor-induced cell division is paralleled by translocation of Gi to the nucleus. FASEB J 5: 200–206. 1900794 10.1096/fasebj.5.2.1900794

[36] Aylin C, Hanyaloglu, Zastrow M (2008) Regulation of GPCRs by Endocytic Membrane Trafficking and Its Potential Implications. Annu Rev Pharmacol Toxicol 48: 537–568. 18184106 10.1146/annurev.pharmtox.48.113006.094830

[37] Re M, Pampillo M, Savard M, Dubuc C, McArdle C, et al (2010) The Human Gonadotropin Releasing Hormone Type I Receptor Is a Functional Intracellular GPCR Expressed on the Nuclear Membrane. PLoS One 5(7): 5. 10.1371/journal.pone.0011489 PMC2900216 20628612

[38] Boivin B, Lavoie C, Vaniotis G, Baragli A, Villeneuve L, et al (2006) Functional β-adrenergic receptor signalling on nuclear membranes in adult rat and mouse ventricular cardiomyocytes. Cardiovasc Res 71: 69–78. 16631628 10.1016/j.cardiores.2006.03.015

[39] Boivin B, Villeneuve L, Farhat N, Chevalier D (2005) Sub-cellular distribution of endothelin signaling pathway components in ventricular myocytes and heart: lack of preformed caveolar signalosomes.. Allen J Mol Cell Cardiol 38: 665–676. 15808843 10.1016/j.yjmcc.2005.02.011

[40] Gobeil F, Bernier S, Vazquez-Tello A, Brault S, Beauchamp M, et al (2003) Modulation of pro-inflammatory gene expression by nuclear lysophosphatidic acid receptor type-1. J Biol Chem 278: 38875–38883. 12847111 10.1074/jbc.M212481200

[41] Gobeil F, Dumont I, Marrache A, Vazquez-Tello A, Bernier S, et al (2002) Regulation of eNOS expression in brain endothelial cells by perinuclear EP(3) receptors.. Circ Res 90: 682–689. 11934836 10.1161/01.res.0000013303.17964.7a

[42] Lu D, Yang H, Shaw G, Raizada M (1998) Angiotensin II-induced nuclear targeting of the angiotensin type 1 (AT1) receptor in brain neurons. Endocrinology 139: 365–375. 9421435 10.1210/endo.139.1.5679

[43] Marrache A, Gobeil F, Bernier S, Stankova J, Rola-Pleszczynski M, et al (2002) Proinflammatory gene induction by platelet-activating factor mediated via its cognate nuclear receptor. J Immunol Methods 169: 6474–6481. 10.4049/jimmunol.169.11.6474 12444157

[44] O’Malley K, Jong Y, Gonchar Y, Burkhalter A, Romano C (2003) Activation of metabotropic glutamate receptor mGlu5 on nuclear membranes mediates intranuclear Ca2+ changes in heterologous cell types and neurons. J Biol Chem 278: 28210–28219. 12736269 10.1074/jbc.M300792200

[45] Bhattacharya M, Peri K, Ribeiro-Da-Sylva A, Almazan G, Shichi H, et al (1999) Localization of functional Prostaglandin E2 receptors EP3 and EP4 in the nuclear envelope. J Biol Chem 274: 15719–15724. 10336471 10.1074/jbc.274.22.15719

[46] Buu N, Hui R, Falardeau P (1993) Norepinephrine in neonatal rat ventricular myocytes: association with the cell nucleus and binding to nuclear α1- and β-adrenergic receptors. J Mol Cell Cardiol 25: 1037–1046. 8283467 10.1006/jmcc.1993.1116

[47] Gobeil F, Zhu T, Brault S, Geha A, Vazquez-Tello A, et al (2006) Nitric oxide signaling via nuclearized endothelial nitric-oxide synthase modulates expression of the immediate early genes iNOS and mPGES-1. The Journal of Biological Chemistry 281: 16058–16067. 16574649 10.1074/jbc.M602219200

[48] Lind G, Cavanagh H (1995) Identification and subcellular distribution of muscarinic acetylcholine receptor-related proteins in rabbit corneal and Chinese hamster ovary cells. Invest Ophthalmol Vis Sci 36: 1492–1507. 7601630

[49] Miguel B, Calcerrada M, Martin L, Catalan R, Martinez A (2001) Increase of phosphoinositide hydrolysis and diacylglycerol production by PAF in isolated rat liver nuclei.. Prostaglandins Other Lipid Mediat 65: 159–166. 11444588 10.1016/s0090-6980(01)00124-1

[50] Nielsen C, Campbell J, Ohd J, Morgelin M, Riesbeck K, et al (2005) A novel localization of the G-protein-coupled CysLT1 receptor in the nucleus of colorectal adenocarcinoma cells. Cancer Res 65: 732–742. 15705869

[51] Tadevosyan A, Vaniotis G, Allen B, Hebert T, Nattel S (2012) G protein-coupled receptor signalling in the cardiac nuclear membrane: evidence and possible roles in physiological and pathophysiological function. The journal of physiology 590: 1313. 22183719 10.1113/jphysiol.2011.222794 PMC3382322

[52] Boivin B, Chevalier D, Villeneuve L, Rousseau E, Allen BG (2003) Functional endothelin receptors are present on nuclei in cardiac ventricular myocytes. The Journal of Biological Chemistry 278: 29153–29163. 12756260 10.1074/jbc.M301738200

[53] Mehta T, Lu H, Wang X, Urvalek A, Nguyen K, et al (2009) A unique sequence in the N-terminal regulatory region controls the nuclear localization of KLF8 by cooperating with the C-terminal zinc-fingers. Cell Research 19: 1098–1109. 19488069 10.1038/cr.2009.64

[54] McLane LM, Corbett AH (2009) Nuclear localization signals and human disease. IUBMB Life 61: 697–706. 19514019 10.1002/iub.194

[55] Poon I, Jans D (2005) Regulation of nuclear transport: central role in development and transformation? Traffic 6: 173–186. 15702986 10.1111/j.1600-0854.2005.00268.x

[56] Lee S, Hannink M (2003) Molecular mechanisms that regulate transcription factor localization suggest new targets for drug development. Adv Drug Deliv Rev 55: 717–731. 12788536 10.1016/s0169-409x(03)00052-8

[57] Weis K (2003) Regulating access to the genome: nucleocytoplasmic transport throughout the cell cycle. Cell 112: 441–451. 12600309 10.1016/s0092-8674(03)00082-5

[58] King MC, Lusk CP, Blobel G (2006) Karyopherin-mediated import of integral inner nuclear membrane proteins. Nature 442: 1003–1007. 16929305 10.1038/nature05075

[59] Quimby BB, Corbett AH (2001) Nuclear transport mechanisms. Cell Mol Life Sci 58: 1766–1773. 11766877 10.1007/PL00000816 PMC11337321

[60] Lange A, Mills RE, Lange CJ, Stewart M, Devine SE, et al (2007) Classical nuclear localization signals: definition, function, and interaction with importin alpha. J Biol Chem 282: 5101–5105. 17170104 10.1074/jbc.R600026200 PMC4502416

[61] Wang L, Liu Q, Xu B, Chen W, Yang Q, et al (2010) Identification of nuclear localization sequence of CXCR4 in renal cell carcinoma by constructing expression plasmids of different deletants. Plasmid 63: 68–72. 19796655 10.1016/j.plasmid.2009.09.004

[62] Favre N, Camps M, Arod C, Chabert C, Rommel C, et al (2008) Chemokine receptor CCR2 undergoes transportin1-dependent nuclear translocation. Proteomics 8: 4560–4576. 18846510 10.1002/pmic.200800211

[63] Huber J, Cronshagen U, Kadokura M, Marshallsay C (1998) Snurportin1, an m3G-cap-specific nuclear import receptor with a novel domain structure. EMBO J 17: 4114–4126. 9670026 10.1093/emboj/17.14.4114 PMC1170744

[64] Fassati A, Gorlich D, Harrison I, Zaytseva L, Mingot J (2003) Nuclear import of HIV-1 intracellular reverse transcription complexes is mediated by importin 7. EMBO J 22: 3675–3685. 12853482 10.1093/emboj/cdg357 PMC165627

[65] Bridger G, Skerlj R, Thornton D, Padmanabhan S, Martelucci S, et al (1995) Synthesis and structure-activity relationships of phenylenebis(methylene)-linked bis-tetraazamacrocycles that inhibit HIV replication. Effects of macrocyclic ring size and substituents on the aromatic linker. J Med Chem 38: 366–378. 7830280 10.1021/jm00002a019

[66] Mehta T, Lu H, Wang X, Urvalek A, Nguyen K, et al (2009) A unique sequence in the N-terminal regulatory region controls the nuclear localization of KLF8 by cooperating with the C-terminal zinc-fingers. Cell Research 19: 1098–1109. 19488069 10.1038/cr.2009.64

[67] Miroslaw J, Barbara L, Krystyna D (2011) Migratory capabilities of human umbilical cord blood-derived neural stem cells (HUCB-NSC) in vitro. Acta Neurobiol Exp 71: 24–35. 10.55782/ane-2011-1820 21499324

[68] Shang-Chiung W (2010) Nuclear expression of CXCR4 is associated with advanced colorectal cancer. Int J Colorectal Dis 25: 1185–1191. 20607251 10.1007/s00384-010-0999-1

[69] Shibuta K, Mori M, Shimoda K, Inoue H, Mitra P, et al (2002) Regional expression of CXCL12/CXCR4 in liver and hepatocellular carcinoma and cell-cycle variation during in vitro differentiation. Jpn J Cancer Res 93: 789–797. 12149145 10.1111/j.1349-7006.2002.tb01321.x PMC5927066

[70] Wang N, Wu Q, Fang Y, Mai H, Zeng M, et al (2005) Expression of chemokine receptor CXCR4 in nasopharyngeal carcinoma: pattern of expression and correlation with clinical outcome. Journal of Translational Medicine 3: 26. 15978137 10.1186/1479-5876-3-26 PMC1188078

[71] Bai S, Wang D, Klein M, Siegal G (2011) Characterization of CXCR4 expression in chondrosarcoma of bone. Arch Pathol Lab Med 135: 753–758. 21631268 10.5858/2009-0230-OA.1

[72] Wang L, Liu Q, Xu B, Chen W, Yang Q, et al (2010) Identification of nuclear localization sequence of CXCR4 in renal cell carcinoma by constructing expression plasmids of different deletants. Plasmid 63: 68–72. 19796655 10.1016/j.plasmid.2009.09.004

[73] Wang L, Liu Q, Xu B, Chen W, Yang Q, et al (2010) Identification of nuclear localization sequence of CXCR4 in renal cell carcinoma by constructing expression plasmids of different deletants. Plasmid 63: 68–72. 19796655 10.1016/j.plasmid.2009.09.004

[74] Tao M, Kruhlak M, Xia S, Androphy E, Zheng Z (2003) Signals That Dictate Nuclear Localization of Human Papillomavirus Type 16 Oncoprotein E6 in Living Cells. J Virol 77: 13232. 14645580 10.1128/JVI.77.24.13232-13247.2003 PMC296047

[75] Ren M, Qiu S, Venglat P, Xiang D, Feng L, et al (2011) Target of Rapamycin Regulates Development and Ribosomal RNA Expression through Kinase Domain in Arabidopsis. Plant Physiology 155: 1367–1382. 21266656 10.1104/pp.110.169045 PMC3046592

[76] Li H, Tsang C, Watkins M, Bertram P, Zheng X (2006) Nutrient regulates Tor1 nuclear localization and association with rDNA promoter. Nature 442: 1058–1061. 16900101 10.1038/nature05020

[77] Singh R, Lokeshwar B (2011) The IL-8 regulated Chemokine Receptor CXCR7 stimulates EGFR signaling to promote prostate cancer growth. Cancer Res 71: 3268–3277. 21398406 10.1158/0008-5472.CAN-10-2769 PMC3085571

[78] Leung EL, Fiscus RR, Tung JW, Tin VP, Cheng LC, et al (2010) Non-small cell lung cancer cells expressing CD44 are enriched for stem cell-like properties. PLoS One 5: e14062. 21124918 10.1371/journal.pone.0014062 PMC2988826

[79] Hinton CV, Fitzgerald LD, Thompson ME (2007) Phosphatidylinositol 3-kinase/Akt signaling enhances nuclear localization and transcriptional activity of BRCA1. Exp Cell Res 313: 1735–1744. 17428466 10.1016/j.yexcr.2007.03.008 PMC1939819

[80] Burgess A, Buck M, Krauer K, Sculley T (2006) Nuclear localization of the Epstein–Barr virus EBNA3B protein. Journal of General Virology 87: 789–793. 16528026 10.1099/vir.0.81640-0

[81] Tarasova N, Stauber R, Michejda C (1998) Spontaneous and ligand-induced trafficking of CXC-chemokine receptor 4. J Biol Chem 273: 15883–15886. 9632631 10.1074/jbc.273.26.15883

[82] Zhang Y, Foudi A, Geay J, Berthebaud M, Buet D, et al (2004) Intracellular Localization and Constitutive Endocytosis of CXCR4 in Human CD34+ Hematopoietic Progenitor Cells. Stem Cells 22: 1015–1029. 15536192 10.1634/stemcells.22-6-1015

[83] Chook Y, Blobel G (1999) Structure of the nuclear transport complex karyopherin-b2–Ran×GppNHp. Nature 399: 230–237. 10353245 10.1038/20375

[84] Favre N, Camps M, Arod C, Chabert C, Rommel C, et al (2008) Chemokine receptor CCR2 undergoes transportin1-dependent nuclear translocation. Proteomics 8: 4560–4576. 18846510 10.1002/pmic.200800211

[85] Lu D, Yang H, Shaw G, Raizada M (1998) Angiotensin IIinduced nuclear targeting of the angiotensin type 1 (AT1) receptor in brain neurons. Endocrinology 139: 365–375. 9421435 10.1210/endo.139.1.5679

[86] Zagon I, Ruth T, McLaughlin P (2005) Nucleocytoplasmic distribution of opioid growth factor and its receptor in tongue epithelium. Anat Rec A Discov Mol Cell EvolBiol 282: 24–37. 10.1002/ar.a.20161 15584033

[87] Reilly J, Maher P (2001) Importin {beta}-mediated nuclear import of fibroblast growth factor receptor: Role in cell proliferation. J Cell Biol 152: 1307–1312. 11257130 10.1083/jcb.152.6.1307 PMC2199207

[88] Chook Y, Blobel G (2001) Karyopherins and nuclear import. Curr Opin Struct Biol 11: 703–715. 11751052 10.1016/s0959-440x(01)00264-0

[89] Siomi H, Dreyfus G (1995) A nuclear localization domain in the hnRNP A1 protein. J Cell Biol 129: 551–560. 7730395 10.1083/jcb.129.3.551 PMC2120450

[90] Hamm H (1998) The many faces of G protein signaling. J Biol Chem 273: 669–672. 9422713 10.1074/jbc.273.2.669

[91] Kumar V, Jong Y, Malley K (2008) Activated Nuclear Metabotropic Glutamate Receptor mGlu5 Couples to Nuclear Gq/11 Proteins to Generate Inositol 1,4,5-Trisphosphate-mediated Nuclear Ca2+Release. The Journal of Biological Chemistry 283: 14072–14083. 18337251 10.1074/jbc.M708551200 PMC2376237

[92] Bridger G, Skerlj R, Thornton D, Padmanabhan S, Martellucci S, et al (1995) Synthesis and structure-activity relationships of phenylenebis(methylene)-linked bis-tetraazamacrocycles that inhibit HIV replication. Effects of macrocyclic ring size and substituents on the aromatic linker. J Med Chem 38: 366–378. 7830280 10.1021/jm00002a019

[93] Gobeil F, Fortier A, Zhu T, Bossolasco M, Leduc M, et al (2006) G-protein-coupled receptors signaling at the cell nucleus: an emerging paradigm. Can J Physiol Pharmacol 84: 287–297. 16902576 10.1139/y05-127

[94] Wright C, Chen Q, Baye N, Huang Y, Healy C, et al (2008) Nuclear alpha1-adrenergic receptors signal activated ERK localization to caveolae in adult cardiac myocytes. Circ Res 103: 992–1000. 18802028 10.1161/CIRCRESAHA.108.176024 PMC2792747

[95] Wright C, Wu S, Dahl E, Sazama A, O'Connell T (2012) Nuclear localization drives α1-adrenergic receptor oligomerization and signaling in cardiac myocytes. Cell Signal 24: 794–802. 22120526 10.1016/j.cellsig.2011.11.014 PMC3393107

[96] Marchese A, May M, Temple B, Trejo J (2008) G Protein–Coupled Receptor Sorting to Endosomes and Lysosomes. Annu Rev Pharmacol Toxicol 48: 601–629. 17995450 10.1146/annurev.pharmtox.48.113006.094646 PMC2869288

[97] Barker B, Benovic J (2011) G protein-coupled receptor kinase 5 phosphorylation of hip regulates internalization of the chemokine receptor CXCR4. Biochemistry 50: 6933–6941. 21728385 10.1021/bi2005202 PMC3156627

[98] Pelchen-Matthews A, Signoret N, Klasse P, Fraile-Ramos A, Marsh M (1999) Chemokine receptor trafficking and viral replication. Immunol Rev 168: 33–49. 10399063 10.1111/j.1600-065x.1999.tb01281.x

[99] Jong Y, Kumar V, Kingston A, Romano C, O’Malley K (2005) Functional Metabotropic Glutamate Receptors on Nuclei from Brain and Primary Cultured Striatal Neurons. THE JOURNAL OF BIOLOGICAL CHEMISTRY 280: 30469–30480. 15958386 10.1074/jbc.M501775200

[100] Razani B, Woodman S, Lisanti M (2002) Caveolae: From Cell Biology to Animal Physiology. Pharmacol Rev 54: 431–467. 12223531 10.1124/pr.54.3.431

[101] Kato M, Kitayama J, Kazama S, Nagawa H (2003) Expression pattern of CXC chemokine receptor-4 is correlated with lymph node metastasis in human invasive ductal carcinoma. Breast Cancer Res 144–150. 10.1186/bcr627 PMC314431 12927045

[102] Spano J, Andre F, Morat L, Sabatier L, Besse B, et al (2004) Chemokine receptor CXCR4 and early-stage nonsmall cell lung cancer: pattern of expression and correlation with outcome. Ann Oncol 15: 613–617. 15033669 10.1093/annonc/mdh136

[103] Speetjens F, Liefers G, Korbee C, Mesker W, van de Velde CJH, et al (2009) Nuclear Localization of CXCR4 Determines Prognosis for Colorectal Cancer Patients. Cancer Microenvironment 2: 1–7. 19308676 10.1007/s12307-008-0016-1 PMC2787924

[104] Wang S, Lin J, Wang H, Yang S, Li A, et al (2010) Nuclear expression of CXCR4 is associated with advanced colorectal cancer. 25 10: 1185–1191. 10.1007/s00384-010-0999-1 20607251

[105] Yoshitake N, Fukui H, Yamagishi H, Sekikawa A, Fujii S, et al (2008) Expression of SDF-1α and nuclear CXCR4 predicts lymph node metastasis in colorectal cancer. British Journal of Cancer 98: 1682–1689. 18443596 10.1038/sj.bjc.6604363 PMC2391124

[106] Boivin B, Vaniotis G, Allen B, Hebert T (2008) G Protein-Coupled Receptors in and on the Cell Nucleus: A New Signaling Paradigm? Journal of Receptors and Signal Transduction 28: 15–28. 18437627 10.1080/10799890801941889

[107] Valdehita A, Bajo A, Fernández-Martínez A, Arenas M, Vacas E, et al (2010) Nuclear localization of vasoactive intestinal peptide (VIP) receptors in human breast cancer. Peptides 31: 2035–2045. 20691743 10.1016/j.peptides.2010.07.024

[108] Akashi T, Koizumi K, Tsuneyama K, Saiki I, Takano Y, et al (2008) Chemokine receptor CXCR4 expression and prognosis in patients with metastatic prostate cancer. Cancer Sci 99: 539–542. 18201276 10.1111/j.1349-7006.2007.00712.x PMC11158982

[109] Taichman R, Cooper C, Keller E, Pienta K, Taichman N, et al (2002) Use of the stromal cell-derived factor-1/CXCR4 pathway in prostate cancer metastasis to bone. Cancer Res 62: 1832–1837. 11912162

[110] Sun Y, Wang J, Shelburne C, Lopatin D, Chinnaiyan A, et al (2003) Expression of CXCR4 and CXCL12 (SDF-1) in human prostate cancers (PCa) in vivo. J Cell Biochem 89: 462–473. 12761880 10.1002/jcb.10522

[111] Uchida D, Onoue T, Tomizuka Y, Begum N, Miwa Y, et al (2007) Involvement of an autocrine stromal cell derived factor-1/CXCR4 system on the distant metastasis of human oral squamous cell carcinoma. Mol Cancer Res 5: 685–694. 17634424 10.1158/1541-7786.MCR-06-0368

[112] Dessein A, Stechly L, Jonckheere N, Dumont P, Monté D, et al (2010) Autocrine induction of invasive and metastatic phenotypes by the MIF-CXCR4 axis in drug-resistant human colon cancer cells. Cancer Res 70: 4644–4654. 20460542 10.1158/0008-5472.CAN-09-3828

[113] Woo S, Bae J, Kim C, Lee J, Koo B (2008) A Significant Correlation between Nuclear CXCR4 Expression and Axillary Lymph Node Metastasis in Hormonal Receptor Negative Breast Cancer. Annals of Surgical Oncology 15: 281–285. 17763975 10.1245/s10434-007-9595-1

[114] Chelsky D, Ralph R, Jonak G (1989) Sequence requirement for synthetic peptide mediated translocation to the nucleus. Mol Cell Biol 9: 2487–2492. 2668735 10.1128/mcb.9.6.2487-2492.1989 PMC362321

[115] LaCasse E, Lefebvre Y (1995) Nuclear localization signals overlap DNA- or RNA-binding domains in nucleic acid-binding proteins. Nucleic Acids Res 23: 1647–1656. 7540284 10.1093/nar/23.10.1647 PMC306917

[116] Michael W, Eder P, Dreyfuss G (1997) The K nuclear shuttling domain: a novel signal for nuclear import and nuclear export in the hnRNP K protein. EMBO J 16: 3587–3598. 9218800 10.1093/emboj/16.12.3587 PMC1169983

[117] Pollard W, Michael W, Nakielny S (1996) A novel receptor-mediated nuclear protein import pathway. Cell 86: 985–994. 8808633 10.1016/s0092-8674(00)80173-7

[118] Fan X, Steitz J (1998) HNS, a nuclear-cytoplasmic shuttling sequence in HuR. Proc Natl Acad Sci USA 95: 15293–15298. 9860962 10.1073/pnas.95.26.15293 PMC28036

[119] Takei Y, Yamamoto K, Tsujimoto G (1999) Identification of the sequence responsible for the nuclear localization of human Cdc6. FEBS Lett 447: 292–296. 10214964 10.1016/s0014-5793(99)00306-3

[120] Hall M, Craik C, Hiraoka Y (1990) Homeodomain of yeast repressor alpha 2 contains a nuclear localization signal. Proc Natl Acad Sci USA 87: 6954–6958. 1976249 10.1073/pnas.87.18.6954 PMC54660

[121] Ishidate T, Yoshihara S, Kawasaki Y (1997) Identification of a novel nuclear localization signal in Sam68. FEBS Lett 409: 237–241. 9202153 10.1016/s0014-5793(97)00455-9

[122] Burgess A, Buck M, Krauer K, Sculley T (2006) Nuclear localization of the Epstein–Barr virus EBNA3B protein. Journal of General Virology 87: 789–793. 16528026 10.1099/vir.0.81640-0

[123] Chook Y, Blobel G (1999) Structure of the nuclear transport complex karyopherin-beta2-Ran x GppNHp. Nature 399: 230–237. 10353245 10.1038/20375

[124] Chook Y, Blobel G (2001) Karyopherins and nuclear import. Curr Opin Struct Biol 11: 703–715. 11751052 10.1016/s0959-440x(01)00264-0

[125] Reilly J, Maher P (2001) Importin beta-mediated nuclear import of fibroblast growth factor receptor: role in cell proliferation. J Cell Biol 152: 1307–1312. 11257130 10.1083/jcb.152.6.1307 PMC2199207

[126] Baldassare J, Jarpe M, Alferes L, Raben D (1997) Nuclear Translocation of RhoA Mediates the Mitogen-induced Activation of Phospholipase D Involved in Nuclear Envelope Signal Transduction. The Journal Of Biological Chemistry 272: 4911–4914. 9030550 10.1074/jbc.272.8.4911

[127] Lepretre N, Mironneau J, Morel J (1994) Both alpha 1A- and alpha 2A-adrenoreceptor subtypes stimulate voltage- operated L-type calcium channels in rat portal vein myocytes. Evidence for two distinct transduction pathways. J Biol Chem 269: 29546–29552. 7961939

[128] Malviya A, Rogue P (1998) ‘Tell me where is calcium bred’: clarifying the roles of nuclear calcium. Cell 92: 17–23. 9489696 10.1016/s0092-8674(00)80895-8

[129] Berridge M (2001) The versatility and complexity of calcium signalling. Novartis Found Symp 239: 52–64. 11529316

[130] Power J, Sah P (2002) Nuclear Calcium Signaling Evoked by Cholinergic Stimulation in Hippocampal CA1 Pyramidal Neurons. J Neurosci 22: 3454–3462. 11978822 10.1523/JNEUROSCI.22-09-03454.2002 PMC6758367

[131] Gerasimenko O, Gerasimenko J, Tepikin A, Petersen O (1995) ATP-dependant accumulation and inositol triphosphate- or cyclic ADP- ribose-mediated release of Ca2+from the nuclear envelope. 80: 439–444. 10.1016/0092-8674(95)90494-8 7859285

[132] Alonso M, Villalobos C, Chamero P, Alvarez J, Garcia-Sancho J (2006) Calcium microdomains in mitochondria and nucleus. Cell Calcium 40: 513–525. 17067669 10.1016/j.ceca.2006.08.013

[133] Humbert J, Matter N, Artault J, Koppler P, Malviya A (1996) Inositol 1,4,5-trisphosphate receptor is located to the inner nuclear membrane vindicating regulation of nuclear calcium signaling by inositol 1,4,5-trisphosphate. Discrete distribution of inositol phosphate receptors to inner and outer nuclear membranes. J Biol Chem 271: 478–485. 8550605 10.1074/jbc.271.1.478

[134] Dolmetsch R, Lewis R, Goodnow C, Healy J (1997) Differential activation of transcription factors induced by Ca2+response amplitude and duration. Nature 386: 855–858. 9126747 10.1038/386855a0

[135] Lee T, Seng S, Sekine M, Hinton C, Fu Y, et al (2007) Vascular Endothelial Growth Factor Mediates Intracrine Survival in Human Breast Carcinoma Cells through Internally Expressed VEGFR1/FLT1. PLoS Med 4: e186. 17550303 10.1371/journal.pmed.0040186 PMC1885450

